# Spatiotemporal trends in burden of uterine cancer and its attribution to body mass index in 204 countries and territories from 1990 to 2019

**DOI:** 10.1002/cam4.4608

**Published:** 2022-02-13

**Authors:** Songbo Li, Hui Chen, Tongchao Zhang, Rongrong Li, Xiaolin Yin, Jinyu Man, Qiufeng He, Xiaorong Yang, Ming Lu

**Affiliations:** ^1^ Clinical Epidemiology Unit Qilu Hospital of Shandong University Jinan Shandong China; ^2^ Clinical Research Center of Shandong University, Qilu Hospital Cheeloo College of Medicine, Shandong University Jinan Shandong China; ^3^ Department of Epidemiology and Health Statistics, School of Public Health Cheeloo College of Medicine, Shandong University Jinan Shandong China; ^4^ Department of Obstetrics and Gynecology Qilu Hospital of Shandong University Jinan Shandong China

**Keywords:** disease burden, global analysis, high body mass index, temporal trend, uterine cancer

## Abstract

**Background:**

Uterine cancer is one of the most common female cancers worldwide, with huge heterogeneity in morbidity and mortality. Although a high body‐mass index (BMI) has been linked to uterine cancer, systematic reports about the influence of high BMI and its temporal trends are scarce.

**Methods:**

The annual morbidity, mortality, and disability‐adjusted life years (DALYs) of uterine cancer in 204 countries or territories were retrieved from the GBD 2019 study. To reflect trends in disease burden, we also calculated the estimated annual percentage change (EAPC) based on the age‐standardized rates of uterine cancer from 1990 to 2019.

**Results:**

The global incident cases of uterine cancer increased 2.3 times from 187,190 in 1990 to 435,040 in 2019. Although the age‐standardized incidence rate (ASIR) of uterine cancer increased worldwide from 8.67/100,000 in 1990 to 9.99/100,000 in 2019, the age‐standardized death rate (ASDR) and DALY rate decreased during the same period. High socio‐demographic index (SDI) countries tended to have a higher ASIR than developing regions, and their increasing trend in ASIR was also more pronounced. The disease was rare before 40 years old, but its risk rose sharply among women aged 50–70. A high BMI was linked to more than one‐third of deaths from uterine cancer in 2019.

**Conclusions:**

The incidence in developed areas was significantly higher than in developing areas and also increased much more rapidly. Elderly females, especially those with a high BMI, have a higher risk of uterine cancer. Therefore, more health resources may be needed to curb the rising burden in specific populations.

## Graphical abstract

The uterine cancer incidence in developed areas is significantly higher than that in developing areas and also increased much more rapidly. Elderly females, especially those with a high BMI, had a higher risk of uterine cancer. Therefore, more health resources may be needed to curb the rising burden of specific populations.

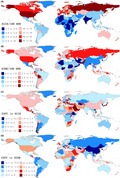

## 1 INTRODUCTION

Global cancer incidence and mortality are rising rapidly,^1^ with nearly 19.3 million new cases and 10.0 million deaths worldwide in 2020.^2^ This increase in the overall burden of uterine cancer is believed to result from population growth, aging, and changes in major risk factors, some of which are related to socio‐economic development.^1^, ^2^ Globally, uterine cancer is a top‐fifth female cancer with high death rates,^3^, ^4^ and the corresponding incidence and death rates vary widely across the world.^1^, ^5^ Hence, understanding geographic and temporal trends in uterine cancer burden at the global, regional, and national levels is essential for policy development and rational resource allocation.

Although the etiology of uterine cancer is still unknown, several risk factors have been identified, such as obesity,^6^ metabolic syndrome, diabetes mellitus,^7^, ^8^ and hypertension,^9^ which may explain variations in different regions. Few studies have analyzed and estimated the global burden of uterine cancer due to various etiologies.^10^ Previous studies on the disease burden of uterine cancer have been based on data from several countries, including Egypt,^11^ American Samoa,^12^ and Southern Thailand.^13^ The Global Burden of Disease (GBD) Study is a comprehensive database that gives the annual morbidity, mortality, and disability‐adjusted life years (DALYs) rates due to uterine cancer and its risk factors, by using robust statistical methods to analyze data from 204 countries and territories. This study analyzed these data from 1990 to 2019, the first such study to do so. The aim of this study was to evaluate patterns in the disease burden of uterine cancer and determine the contribution of high BMI, which is essential for policy development and prevention efforts.

## 2 MATERIALS AND METHODS

### 2.1 Data sources

The detailed data of incidence, mortality, DALYs, and the corresponding age‐standardized rates (ASRs) of uterine cancer were collected from the GBD 2019 study via the Global Health Data Exchange (GHDx) website (http://ghdx.healthdata.org/gbd‐results‐tool). These data originated from multiple cancer databases, such as Cancer Incidence in Five Continents (CI5), NORDCAN, and SEER. The flows and modeling codes for GBD study analysis can be accessed through the following address: http://ghdx.healthdata.org/gbd‐2019/code. Based on geographic units or locations, the GBD 2019 database contains 21 regions nested within seven super‐regions, and there were 204 countries or territories within the 21 regions.

We used the latest SDI to determine the relationship between a country's level of health development and uterine cancer incidence, mortality, and DALY rates. To report on aggregate results, geographies were divided into SDI quintiles as high, high‐middle, middle, low‐middle, and low SDI regions. Quintile cutoffs were based on the entire distribution of geography–years from 1990 to 2019, excluding populations smaller than 1 million. The SDI values range from 0 (worst) to 1 (best), reflecting the degree of health development according to resident income per capita, educational attainment, and total fertility rate.^14^ The comparative risk assessment (CRA) framework was used to assess the proportion of DALYs of uterine cancer attributable to high BMI^15^: Identify convincing risk factors with relative risk based on the systematic reviews and meta‐regression, assess the exposure levels and distributions by spatiotemporal Gaussian regression and Bayesian meta‐regression methods, preset the theoretical minimal exposure risk level, and estimate the population attributable fractions (PAFs).

### 2.2 Evaluation of uterine cancer burden

The incidence and mortality of uterine cancer in the GBD data were determined in the following ways: (1) Based on the data sources that reported incidence of and death from uterine cancer with international disease classification codes, the mortality‐to‐incidence ratio (MIR) was calculated; (2) cancer registry incidence data were multiplied by the MIR to calculate mortality estimates; (3) all of these data were used as input to follow the Cause of Death Ensemble model process to determine the cancer‐specific mortality of uterine cancer; (4) the incidence was generated by dividing the cancer‐specific mortality of uterine cancer estimates by the MIR.^14^ The age‐standardized incidence and mortality rates of uterine cancer were estimated using the GBD 2019 Population Estimates.^16^, ^17^ DALYs were calculated by sex as the sum of years of life lost (YLLs) and years lived with disability (YLDs) for each location, year, age group, and cause.^18^

### 2.3 Statistical analysis

The incidence, mortality, DALYs, and their corresponding ASRs were analyzed to quantify the trends of uterine cancer burden in various regions. To show the changing trends of burden, we also calculated the estimated annual percentage change (EAPC) based on ASRs due to uterine cancer from 1990 to 2019. This was estimated by a regression model fitted to the natural logarithm of the rate, namely ln(rate) = *α* + β*(calendar year) + *ε*.^19^, ^20^, ^21^ EAPC was defined as 100 × (exp [*β*]−1) and the 95% confidence intervals (CIs) of EAPC were included in the fitted model as well.

For the risk factors, the CRA framework was used to estimate the proportion of DALYs attributable to BMI for uterine cancer. CRA was conducted through the following six key steps: (1) Risk‐outcome pairs that satisfied the principle of providing convincing or probable evidence based on researches were included; (2) the relative risk was estimated as a function of exposure based on the systematic reviews and meta‐regression; (3) exposure levels and distributions were estimated by using the spatiotemporal Gaussian process regression, DisMod‐MR 2.1, a Bayesian meta‐regression method, and other methods; (4) the theoretical minimum risk exposure level was defined as the exposure level associated with minimum risk determined from published trials and cohort studies; (5) the population attributable fractions (PAFs) and attributable burden were calculated; (6) PAFs and attributable burden for combinations of risk factors were estimated by considering the mediation of different risk factors through other risk factors.^15^, ^22^

The 87 risk factors from the GBD study were divided into four main types: Environmental and occupational, metabolic, behavioral and dietary risks. The risk factors for various diseases in the GBD study were determined by the GBD Collaborators according to the convincing or possible evidence classification of the World Cancer Research Fund. Among them, there is sufficient evidence to show the relationship between high BMI and uterine cancer development, and the comparative risk assessment framework in the GBD study is used to estimate the attribution ratio of high BMI to the potential burden of disease.^23^, ^24^ In brief, the framework of comparative risk assessment included the following steps: Identifying strong risk‐outcome pairs, estimating relative risks, assessing exposure levels and distributions, determining the theoretical minimum exposure level, calculating the population attributable proportion and attributable burden, and estimating the combined risk factor attributable proportion considering the mediating effect.^15^, ^17^

We used Spearman rank correlation to quantify the relationship between the EAPCs in uterine cancer burden and the baseline burden in 1990 and the SDI in 2019 at the national level. The age‐standardized rate of uterine cancer burden in 1990 could be used as a proxy for the baseline disease reservoir, and the SDI in 2019 represents the level and availability of health care in each country.

All statistical analyses in the current study were conducted using R v. 4.0.3 (https://www.R‐project.org/), and a two‐sided *p* value <0.05 was considered statistically significant.

## 3 RESULTS

### 3.1 The current burden of uterine cancer and its changing trend

The global number of incident cases of uterine cancer increased 2.3 times from 187,190 (95% uncertainty interval [UI]: 174,630, 196,030) cases in 1990 to 435,040 (397,020, 479,730) cases in 2019 (Table S1). The EAPC in ASIR was 0.69 (95% CI: 0.57, 0.81), from 8.67 (95% UI: 8.10, 9.08)/100,000 in 1990 to 9.99 (9.12, 11.02)/100,000 in 2019 (Table S1). Uterine cancer was responsible for 91,640 (95% UI: 82390, 101,500) deaths globally in 2019. The global death toll increased 1.6 times from 56,130 (51,100, 60,200) in 1990 to 91,640 (95% UI: 82390, 101,500) in 2019 (Table S2). The global ASDR was 2.67 (95% UI: 2.44, 2.86)/100,000 in 1990, which decreased to 2.09 (1.88, 2.32)/100,000 in 2019 (EAPC: −0.85 [95% CI: −0.93, −0.76); Table S2). Uterine cancer was responsible for 2.33 million (95% UI: 2.09, 2.56) DALYs globally, with an age‐standardized DALY rate of 53.54 (95% UI: 48.13, 58.84)/100,000 in 2019. This was a 1.4‐fold increase from 1.48 million (95% UI, 1.32, 1.61) DALYs in 1990, but the age‐standardized DALY rate decreased from 1990 to 2019 (EAPC: −0.84 [95% CI, −0.93, −0.75]; Table S3).

**FIGURE 1:**
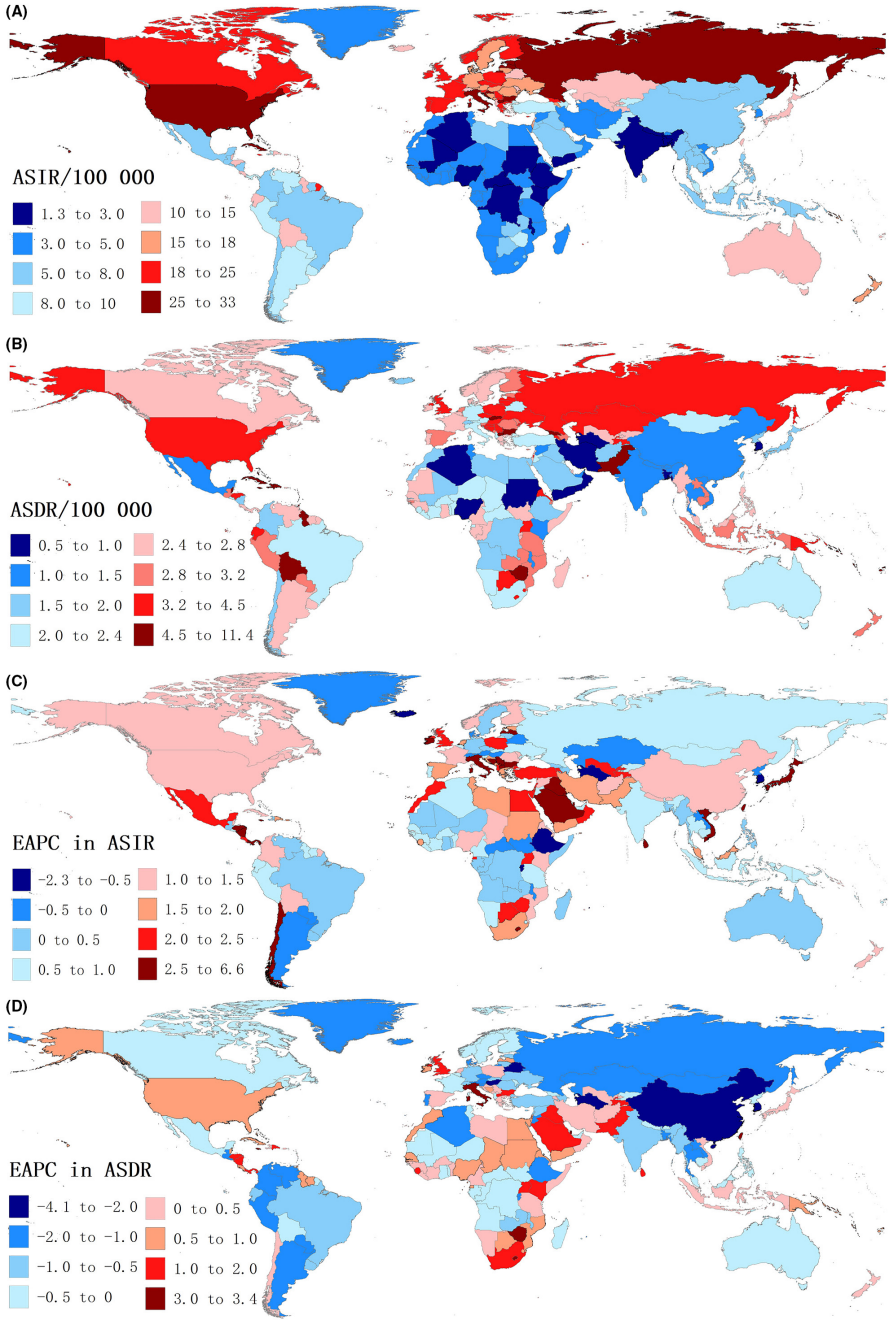
The global disease burden of uterine cancer in 204 countries and territories. (A) ASIR of uterine cancer in 2019, (B) ASDR of uterine cancer in 2019, (C) EAPC in ASIR of uterine cancer in 2019, and (D) EAPC in ASDR of uterine cancer in 2019. ASDR, age‐standardized death rate; ASIR, age‐standardized incidence rate; EAPC, estimated annual percentage change

In 2019, the highest ASIR for uterine cancer was in Northern Mariana Islands (32.77 [95% UI 21.30, 42.36]/100,000), followed by Russia (32.55/100,000), and Bulgaria (30.66/100,000) (Figure 1A; Table S5). The lowest ASIR in 2019 was found in Nigeria (1.31 [0.80, 2.83]/100,000), and the ASIR in 15 other countries and regions including Bangladesh, Palau, and Yemen was less than 3/100,000 (Figure 1A). The ASIR in Northern Mariana Islands was nearly 25 times higher than that in Nigeria. Moreover, Taiwan (EAPC: 6.57 [95% CI: 6.02, 7.13]), Italy (4.81[95% CI: 4.10, 5.53]), and Saudi Arabia (4.76 [95% CI: 4.47, 5.05]) showed the largest increases in ASIR (Figure 1C; Table S7).

In 2019, ASDRs were highest in Grenada (11.30 [9.79–12.99]/100,000), American Samoa (10.74/100,000), and Saint Vincent and the Grenadines (8.08/100,000) (Figure 1B; Table S6). Conversely, Palau (0.52/100,000), Algeria (0.75/100,000), and South Korea (0.77/100,000) had the lowest ASDRs in 2019 (Figure 1B). Taiwan (EAPC: 3.38 [95% CI: 2.92, 3.85]), Lesotho (3.27 [2.81, 3.74]), and Jamaica (3.21 [2.83, 3.60]) showed the largest increases in ASDR (Figure 1D; Table S8). Figure 1A demonstrates that countries with high ASIR are mainly concentrated in North America and Eastern Europe. While countries with high ASDR are mostly concentrated in Oceania and the Caribbean, many countries in Eastern Europe have high incidence and death rates. Besides, the geographic distribution of the age‐standardized DALY rate was highly similar to ASDRs. DALY data for specific countries and regions are provided in the appendix (Figure S1; Table S3).

### 3.2 The correlation between SDI and the age‐standardized incidence, death rate and DALY rates of uterine cancer

**FIGURE 2:**
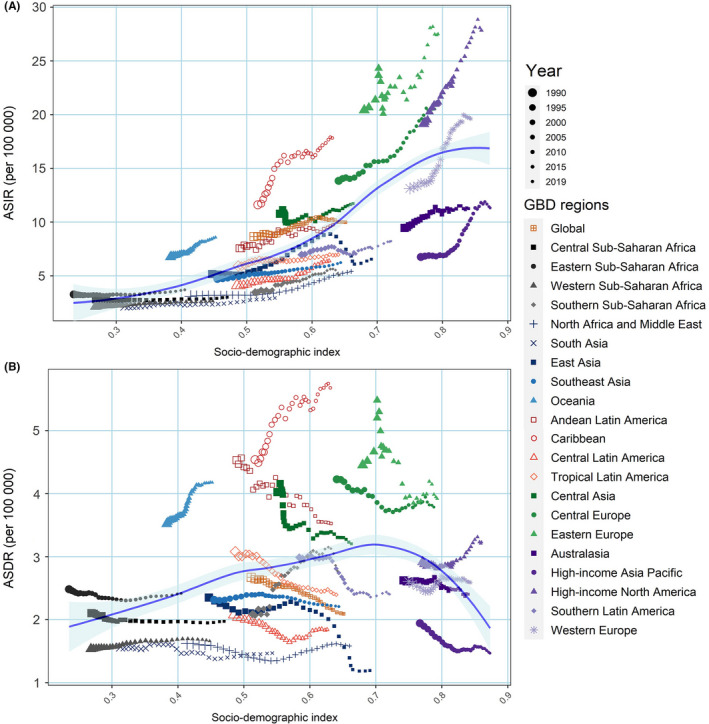
The change trends and correlation analyses of ASRs and SDI from 1990 to 2019. Expected values based on SDI and ASRs in all locations are shown as the blue line. For each region, points from big to small depict estimates from each year from 1990 to 2019. (A) The change trends and correlation of ASIR and SDI from 1990 to 2019 in 21 regions. (B) The change trends and correlation of ASDR and SDI from 1990 to 2019 in 21 regions. ASDR, age‐standardized death rate; ASIR, age‐standardized incidence rate; DALY, disability‐adjusted life year; SDI, socio‐demographic index

We investigated the association between SDI and ASIR, ASDR and age‐standardized DALY rate in 21 GBD regions (Figure 2). The high SDI regions with relatively heavy burdens presented a more pronounced increase in ASIR from 1990 to 2019 than many other regions, such as North America (EAPC: 1.44 [95% CI: 1.34, 1.53]) and Western Europe (1.71 [1.57, 1.86]; Figure 2A; Table S1). However, the ASDR is relatively low in these high SDI regions, such as Western Europe (ASDR: [2.59/10,000]; EAPC 0.2 [0.10, 0.31]), and has not changed much over the past 30 years. In addition, Western Sub‐Saharan Africa had the lowest ASIR in 2019, and this remained relatively stable from 1990 (ASIR: [2.64/10,000]; EAPC: 0.83 [0.77, 0.89]; Table S1; Figure 2A). Figure 2B demonstrates that ASDR in most regions has been declining from 1990 to 2019, while the Caribbean (5.68/100,000) and Oceania (4.18/100,000) not only had the highest ASDR in 2019, but also had an increasing trend from 1990 to 2019 (Table S2; Figure 2B). By contrast, East Asia (1.19/100,000), South Asia (1.47/100,000), and High‐income Asia Pacific (1.47/100,000) had the lowest ASDR in 2019 (Table S2; Figure 2B). The relationship between SDI and age‐standardized DALY rate in 21 GBD regions was highly similar to ASDR. Specific data for DALYs can be found in Appendix (Table S3; Figure S2).

**FIGURE 3:**
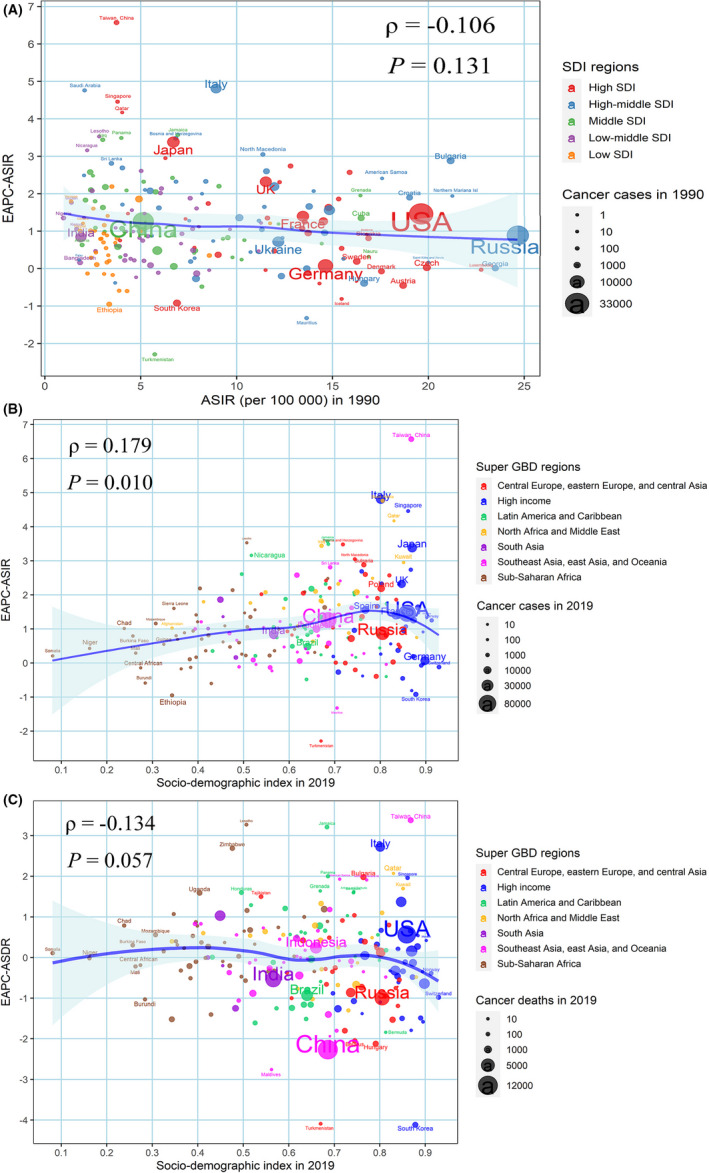
The factors affected the EAPCs in age‐standardized burden rate of uterine cancer from 1990 to 2019, both sexes, at the national level. (A) ASIR of uterine cancer in 1990 and EAPC in ASIR (B) SDI in 2019 and EAPC in ASIR; (C) SDI in 2019 and EAPC in ASDR. The circles represent countries and the size of the circle is increased with the number of uterine cancer patients. The ρ indices and *p* values presented were derived from Spearman rank analysis. The blue line and its shade were fitted by LOESS. The blue line represents the average expected relationship between SDI and burden estimates rates for uterine cancer based on values from each geographical region over the 1990–2019 estimation period. Shading indicates the upper and lower limits of the 95% confidence intervals (CIs). ASDR, age‐standardized death rate; ASIR, age‐standardized incidence rate; EAPC, estimated annual percentage change; SDI, socio‐demographic index

The EAPC of ASIR from 1990 to 2019 varied significantly among the GBD regions, and the major causes may be the baseline disease burden in 1990 and the latest SDI of each country or territory. First, the correlation coefficient between ASIR in 1990 and the corresponding EAPC value was calculated. The results showed that there were no statistically significant correlations between the EAPC of ASIR and ASIR in 1990 (*ρ* = −0.106, *p* = 0.131, respectively), and it indicated that countries with a heavy burden of uterine cancer may not pay enough attention to its prevention and management (Figure 3A). We also evaluated the association between SDI in 2019 and EAPC of ASIR, ASDR, and age‐standardized DALY rate. It was found that the EAPC of ASIR was positively correlated with SDI in 2019 (Figure 3B, *ρ* = 0.179, *p* < 0.05). Moreover, 176 out of 204 countries or territories showed an increase in ASIR from 1990 to 2019. We found no statistically significant association between SDI in 2019 and EAPC of ASDR (Figure 3C); and the EAPC of age‐standardized DALY rate showed a similar pattern (Figure S3).

### 3.3 The incidence, death rate and DALYs of uterine cancer and age structure

**FIGURE 4:**
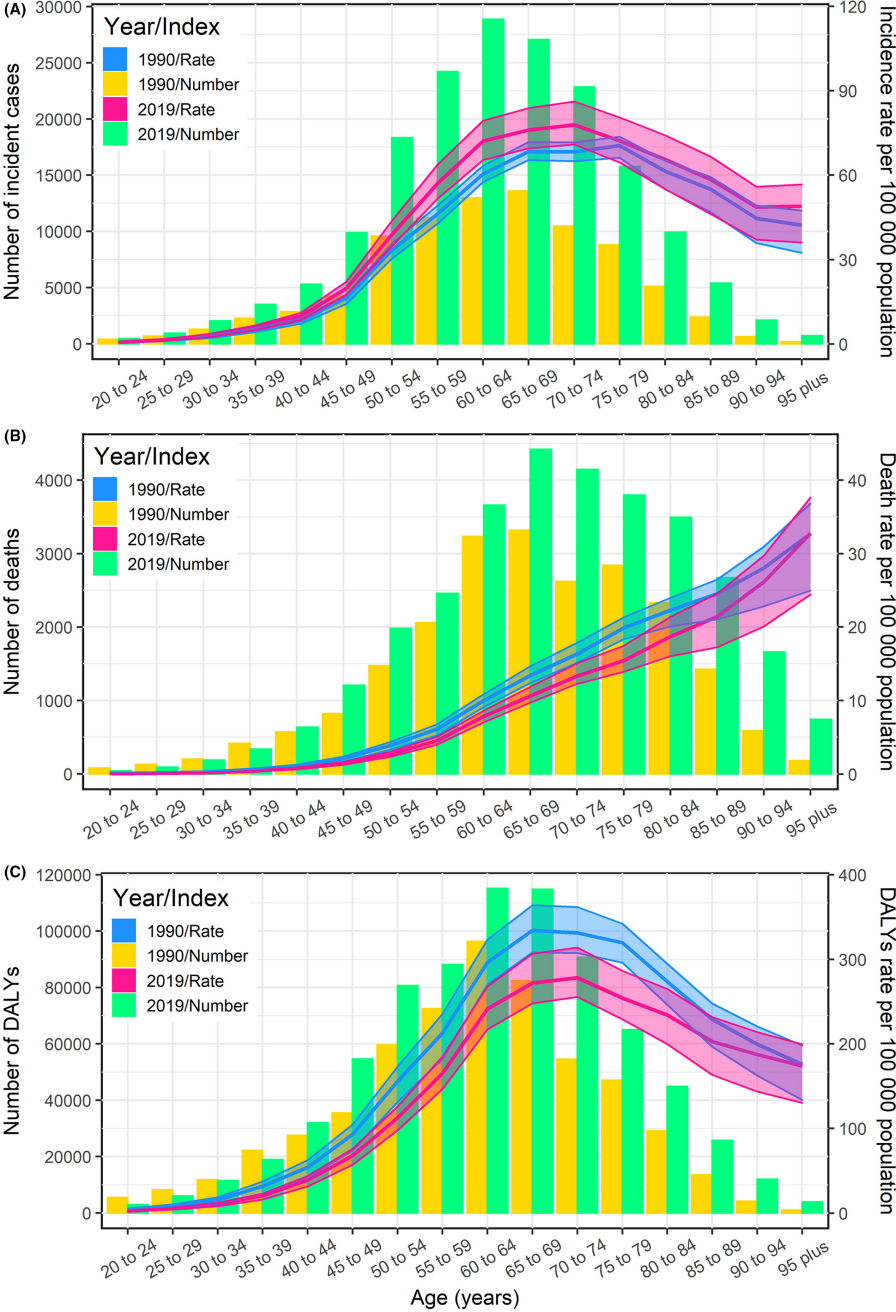
Global number of incidence (A), death (B), DALY (C) cases and incidence (A), death (B), DALY (C) rate of uterine cancer per 100,000 person‐years by age and year (1990 and 2019). Error bars indicate the 95% uncertainty interval for incident cases. The fitted lines represent age‐specific rates of uterine cancer burden by sex in 1990(blue) and 2019(red), and shading indicates the 95% uncertainty interval for the incidence rate. DALY, disability‐adjusted life year; SDI, socio‐demographic index

The disease was rare before the age of 40 years, but risk rose sharply among women in their late 50s to middle 70s. The number of incident cases showed an N‐shaped distribution and peaked at the ages of 60–64 years in 2019 (Table S4; Figure 4A). We also observed a similar pattern in DALYs, which indicates that the burden of uterine cancer is the heaviest among those aged 60 to 70, especially in the high SDI region (Figure 4C). Patients in the high‐middle SDI region aged 55 years or older accounted for the largest number of new cases in 2019 (Figure 4A). The incidence rate generally rose with age, and it was noticeably higher in 2019 than in 1990 before the age of 70–74 years (Figure 4A; Table S4). The pattern of DALY rates was similar to the incidence rate, but the rate started decreasing after the age of 70–74 in 2019 (Figure 4C). The death rate increased almost linearly with age, and was higher in 1990 than in 2019 among most age groups (Figure 4B). Death rate was highest in the oldest age group (≥95 years) in both 1990 and 2019 (Figure 4B).

**FIGURE 5:**
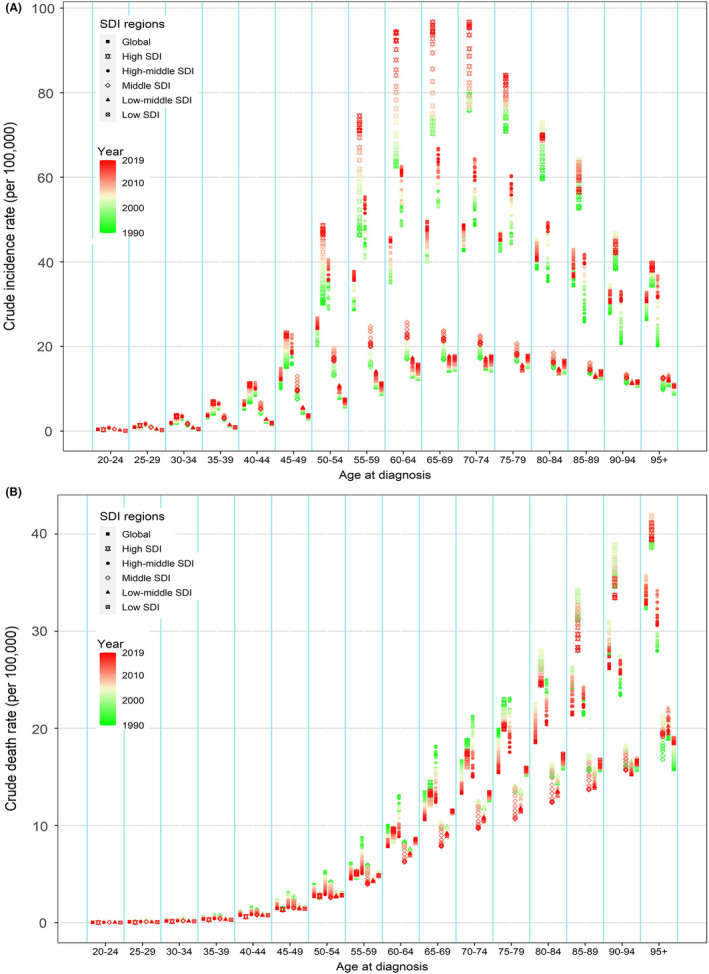
The crude incidence (A) and death (B) rates of uterine cancer in different SDI regions by age from 1990 to 2019. Each age group contains different point symbols, representing different SDI regions, namely global, high, high‐middle, middle, low‐middle and low SDI regions in the order shown in the figure. SDI, socio‐demographic index

We also analyzed trends of uterine cancer incidence, mortality, and DALY rates across age groups in different SDI regions from 1990 to 2019 (Figure 5; Figure S4). The results showed that the crude incidence of uterine cancer was higher in all age groups in the high SDI region in 2019 than in other regions. It also showed a much more obvious increase than other regions from 1990 to 2019, especially after the age of 40 years (Figure 5A). Compared with the high SDI region, the incidence in low SDI countries showed no significant change in different age groups. In addition, among every age group under 60 years, the death rate in different SDI regions showed an obvious decline. After the age of 80 years, no significant decreases in death rates from 1990 to 2019 were found in different SDI regions, and the death rates in both high SDI and high‐middle SDI regions were much higher than in other regions (Figure 5B). The temporal trends of DALY rates were similar to the incidence from 1990 to 2019 (Figure S5).

### 3.4 High BMI is a risk factor for uterine cancer mortality

**FIGURE 6:**
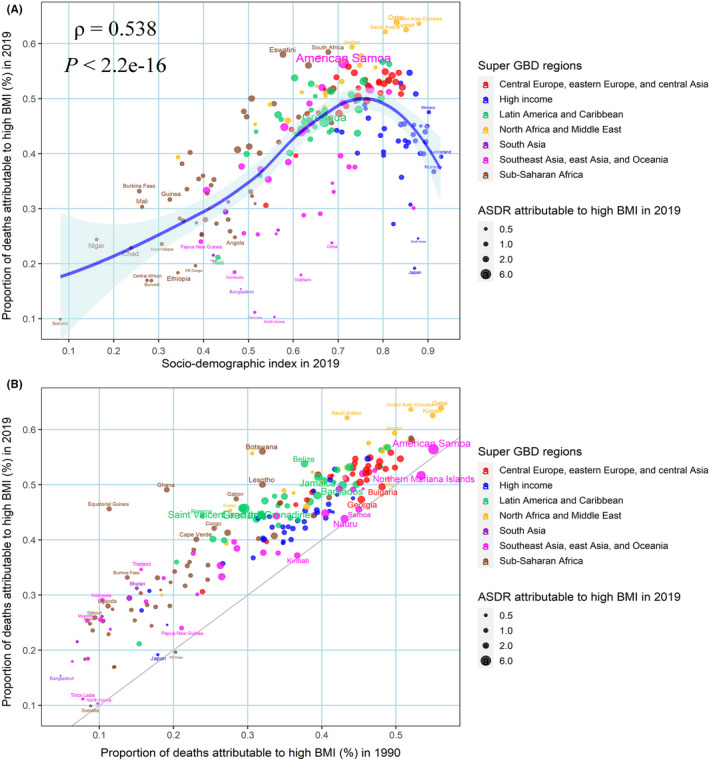
The correlation analyses between the proportion of deaths from uterine cancer attributable to high BMI in 2019 with SDI in 2019 (A) and proportion of deaths attributable to high BMI in 1990 (B). The size of the circle represents the ASDR of uterine cancer attributable to high BMI. ASDR, age‐standardized death rate; BMI, body mass index

The GBD database only identified high BMI as a risk factor for uterine cancer‐related death and DALYs. For countries or territories with different SDI values, it can be observed that high BMI had a significantly higher impact on countries with high SDI than countries with low SDI (Figure 6A). About 36,500 (95% UI 25100–49,200) uterine cancer deaths could be attributed to high BMI in 2019, equivalent to 39.78% (27.62–52.65) of all age‐standardized deaths from uterine cancer. Its contribution ratio rapidly increased from 1990 to 2019 (Figure 6B). In 1990, the proportion of age‐standardized deaths from uterine cancer due to high BMI was 30.70% (19.45–44.05). In 2019, high BMI was linked to the highest proportion of deaths in Qatar (66.07% of all uterine cancer deaths), United Arab Emirates (65.55%) and Saudi Arabia (63.64%). Additionally, the lowest proportion of age‐standardized deaths due to high BMI was found in Democratic People's Republic of Korea (10.35%; Table S9; Figure 6B).

## 4 DISCUSSION

Globally, uterine cancer is a top‐fifth female cancer with high mortality,^3^, ^4^ and is the 14th most common cancer overall. It is broadly classified into Type I and Type II cancer.^25^ Endometrial cancer (EC), a Type I cancer, accounts for approximately 80% of uterine cancer. Type II cancers such as clear cell carcinomas and papillary serous carcinomas account for less than 10%.^26^, ^27^, ^28^, ^29^

In the current study, we analyzed the spatiotemporal trends in burden of uterine cancer over the last three decades. We estimated that there were around 435,040 cases of uterine cancer worldwide, with an ASIR of 9.99/100,000 in 2019. Uterine cancer also accounted for 91,640 total deaths and 2.33 million DALYs in 2019. The ASIR of uterine cancer was significantly higher in some high SDI countries, such as the United States (ASIR: 28.80/100,000; ASDR: 3.30/100,000), which conversely have a relatively low ASDR due to better cancer diagnosis and treatment. The ASIR of uterine cancer in these high SDI countries, such as Italy (EAPC: 4.81), also increased much more than in other regions from 1990 to 2019, which could be attributed to changes in westernization diets and aging of the population in these countries.^30^, ^31^ In fact, obesity and aging are important factors leading to uterine cancer. Conversely, during the same study period, both the age‐standardized death rates and DALY rates declined. According to studies from high SDI countries, various factors may have resulted in this decline, for instance transvaginal ultrasound scanning, improved surgical techniques,^32^, ^33^ chemotherapy, radiotherapy, and targeted therapy.

The increase in ASIR might be due to the introduction of some advanced diagnostic methods, like transvaginal ultrasound scanning or endometrial sampling,^34^ which may have resulted in a higher detection rate and thus increased morbidity. There are significant differences in uterine cancer incidence in different countries, which may be due to variations in exposure to different risk factors or health care levels in different regions.^35^, ^36^ Obesity,^6^ metabolic syndrome, diabetes mellitus,^7^, ^8^ and hypertension^9^ had considerable attributable uterine cancer burden. Obesity is identified as an important risk factor for the development of EC in both pre‐ and postmenopausal women,^37^, ^38^, ^39^, ^40^ and is associated with an increased risk of at least 13 types of cancer, with the highest relative risk for EC.^41^ Overweight and obesity contribute to approximately 39% of the risk of EC.^42^ In Europe, it is estimated that 60% of all new EC cases each year are linked to overweight.^43^ Obesity can also lead to a high estrogen status by increasing the aromatization of estrogen precursors in adipose tissue, which may be the main mechanism linking obesity to EC risk.^43^ Compared with women whose BMI remained the same or slightly increased, women with a lower BMI in later life were 50% less likely to develop EC.^44^ In addition, compared to those who did not lose weight, women who lost weight for more than 5 years had a 25% lower risk of EC,^45^ which demonstrates that weight loss and physical exercise can effectively reduce the uterine cancer risk.

We also investigated the association between sociodemographic factors and uterine cancer‐related mortality. SDI is related to lifestyle, especially dietary patterns, which may contribute to the increase in the above risk factors.^46^ From this study, we found the incidence in high SDI regions was significantly higher than in low SDI regions, and this has increased much more rapidly over the past three decades. For example, uterine cancer is one of the few cancers with rising morbidity and mortality in the United States, partly reflecting the increases in obesity and overweight since the 1980s.^47^

Due to the development of industrialization, xenoestrogens are widely distributed in the environment and have estrogenic effects, which can lead to precocious puberty and menarche.^48^ We found that Taiwan showed the largest increases in both ASIR and ASDR from 1990 to 2019, and the daily average intake of nonylphenol in Taiwan, which acts as a xenoestrogen, was significantly higher than in countries with a low burden of uterine cancer, such as New Zealand and Germany.^49^

The correlation between parity and uterine cancer has been shown in previous studies, which indicates high parity is recognized as a protective factor while low parity is recognized as a risk factor for EC.^50^, ^51^ Egypt, which is considered a low‐middle SDI country, had relatively low incidence and death rates in 2019 (ASIR: 4.45/100,000; ASDR: 1.64/100,000). In 2014, the fertility rate for Egyptian women aged 15–49 years was 3.5 children per woman.^52^ By comparison, the fertility rates in the United States, France, and the United Kingdom were 1.9, 2.0 and 1.9, respectively, from 2011 to 2015, and these countries are all with a relatively high burden of uterine cancer.

In terms of socioeconomic status, the morbidity of uterine cancer was significantly higher in urban areas than in rural areas in China.^53^ As a result of changes in lifestyles and population migration in rural areas, the annual percentage change in rural areas is higher than in urban areas. Therefore, future studies need to investigate the potential reasons for the increased incidence of uterine cancer in high SDI regions.

Moreover, aging is an important factor contributing to uterine cancer. We found that the 70–74 age group had the highest incidence rate (48.69/100,000), which suggests the importance of screening among women of high‐risk ages. This result is similar to research in the United States, where postmenopausal women comprise more than 90% of patients with uterine cancer.^54^ Increased risk of EC is strongly associated with hormone replacement therapy in menopausal women,^51^, ^55^, ^56^ while the combined use of estrogen and progesterone replacement therapy along with oral contraceptive pills reduced the incidence of EC.^38^, ^39^, ^57^ The reduction in the imbalance of exogenous and endogenous hormones may be the reason for the decline in the incidence of uterine cancer among elderly women.^58^

To reduce the heavy burden of uterine cancer, effective prevention strategies needed to be developed to reduce exposure to risk factors, such as limiting the advertisement for and increasing taxes on unhealthy foods, and providing preferential policies to promote the production and consumption of healthy food.^59^, ^60^ Lifestyle changes and physical activity to maintain a healthy BMI may be an effective strategy to reduce EC risk, especially for obese or overweight women.^44^, ^61^, ^62^

In addition, like other cancers, uterine cancer has a much higher chance of survival if caught early and treated more effectively.^63^ Transvaginal ultrasound or endometrial tissue sampling is suitable for the initial assessment of postmenopausal bleeding, and further assessment may be performed by hysteroscopy.^64^ Oral and injectable contraceptives are believed to prevent EC.^65^, ^66^ We noticed from this study that the ASDR is relatively low in high SDI regions compared to areas with low‐ and low‐middle SDI and has not changed much over the past 30 years. These striking disparities show a serious imbalance in health resources between regions, indicating that more attention and investment should be given to previously‐neglected countries or regions.^67^

This study had several limitations, and a major one is the paucity of data on disease burden in some countries. However, various data sources were used for statistical cancer data, such as cancer registries, vital registration systems, as well as epidemiological studies. Advanced statistical modeling methods developed by GBD collaborators somewhat balanced the limitation.

Moreover, the data from the GBD database lacks information on histological types and only contains the risk factor of BMI for uterine cancer. Consequently, several other risk factors for uterine cancer, such as nutritional status, hypertension and diabetes, could not be directly analyzed. However, uterine cancer has a strong association with obesity, and high BMI is positively associated with SDI, which has been proven to be closely related to the burden of uterine cancer in our study. In‐depth research is still needed to improve our understanding of other factors related to the incidence of uterine cancer. Obviously, uterine cancer is still a major challenge to global public health. The results of this study are of great value for the development and implementation of cost‐effective interventions, as well as the reduction of modifiable risk factors.

## 5 CONCLUSIONS

Globally, the incidence of uterine cancer is gradually increasing. Females with high BMI have a higher risk of uterine cancer. The incidence of uterine cancer was pronounced in high‐SDI countries, that is, the incidence in developed countries is significantly higher than in developing countries. Moreover, the incidence has increased rapidly in some developed countries. There is still plenty of space to change lifestyles and increase physical activity to maintain a healthy BMI, especially in developed countries. Overall, given the acceleration of the global aging trend, the number of incident cases and deaths from uterine cancer would further increase. Therefore, public health authorities should reasonably recommend reinforced prevention and management of known and potential major risk factors for uterine cancer to curb the rising burden.

## CONFLICT OF INTEREST

The authors declare that they have no competing interests.

## ETHICAL APPROVAL STATEMENT

The study was reviewed by the ethics committee of Qilu Hospital of Shandong University and approval was obtained (date 28 November 2020, file number KYLL‐202011 [KS]‐239).

## Supporting information


Figure S1

Figure S2

Figure S3

Figure S4

Figure S5

Table S1.
Table S2.
Table S3.
Table S4.
Table S5.
Table S6.
Table S7.
Table S8.
Table S9.

## Data Availability

All data could be download from the online GBD 2019 database.
