# Supplementary figures and images for: Spatiotemporal trends in burden of uterine cancer and its attribution to body mass index in 204 countries and territories from 1990 to 2019
Source: Cancer Med. 2022 Feb 13;11(12):2467–81. doi: 10.1002/cam4.4608 (PMC9189473; doi:10.1002/cam4.4608)

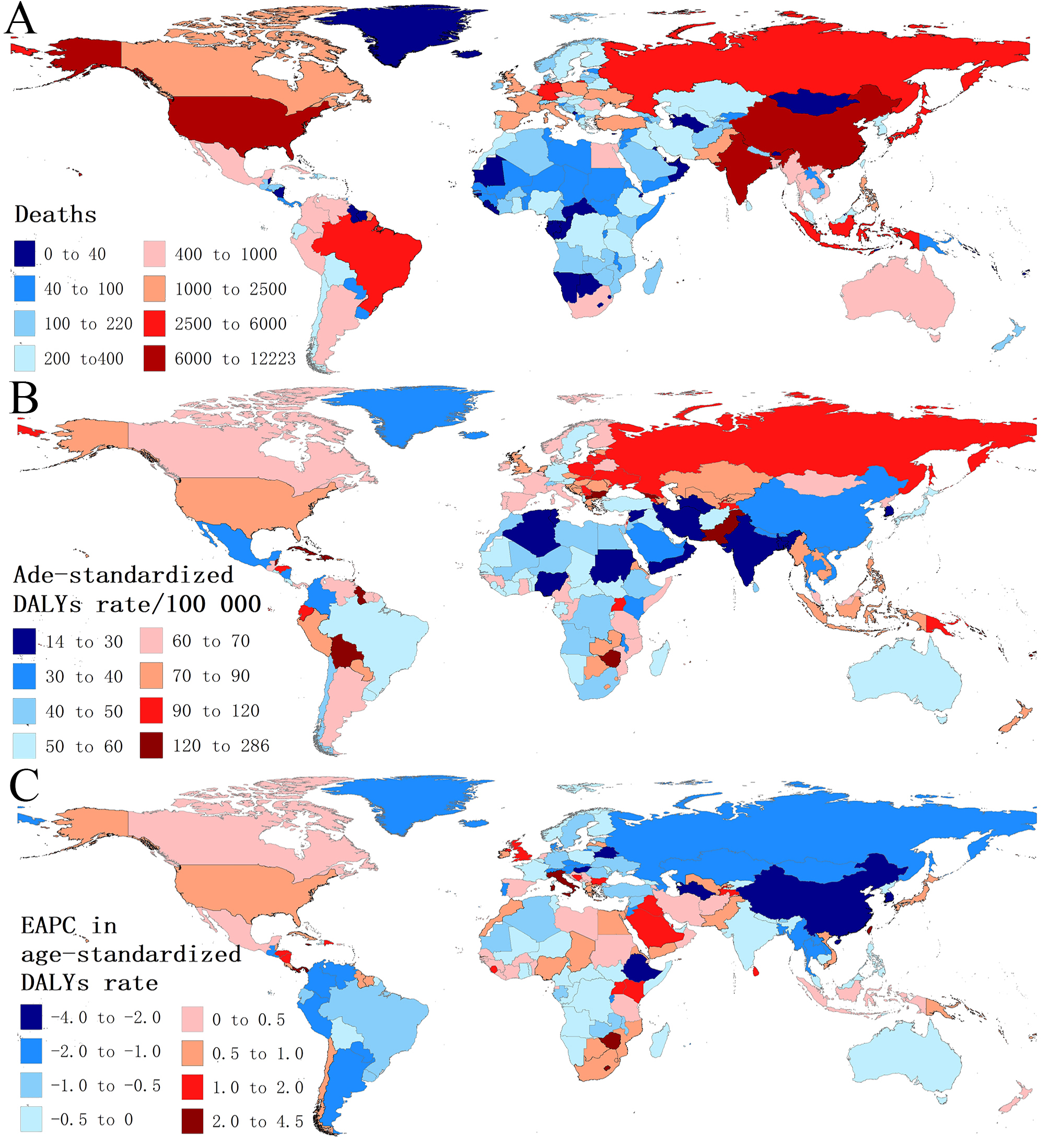

Supplement: Supplementary file 1 — Figure S1 [file CAM4-11-2467-s004.jpg]

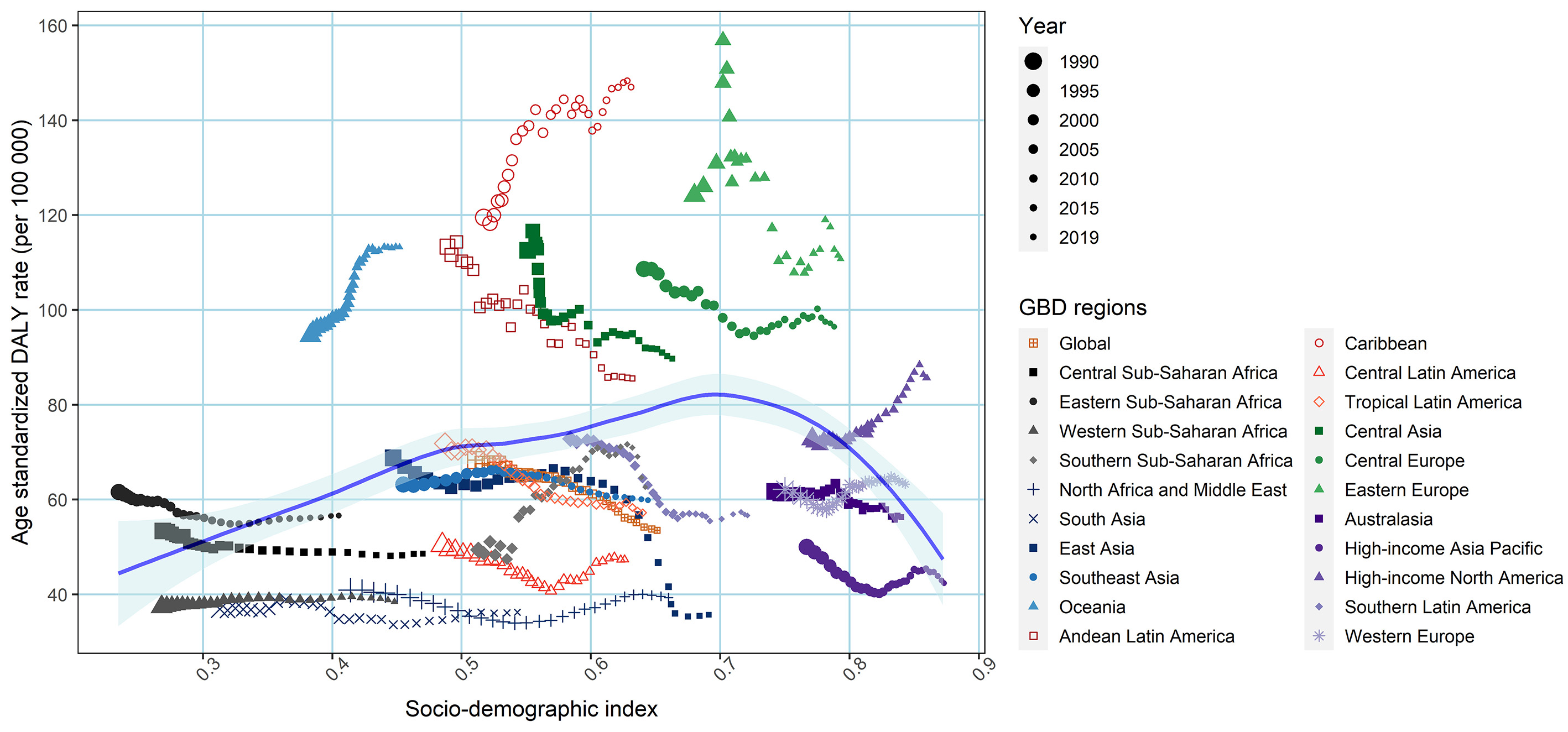

Supplement: Supplementary file 2 — Figure S2 [file CAM4-11-2467-s003.jpg]

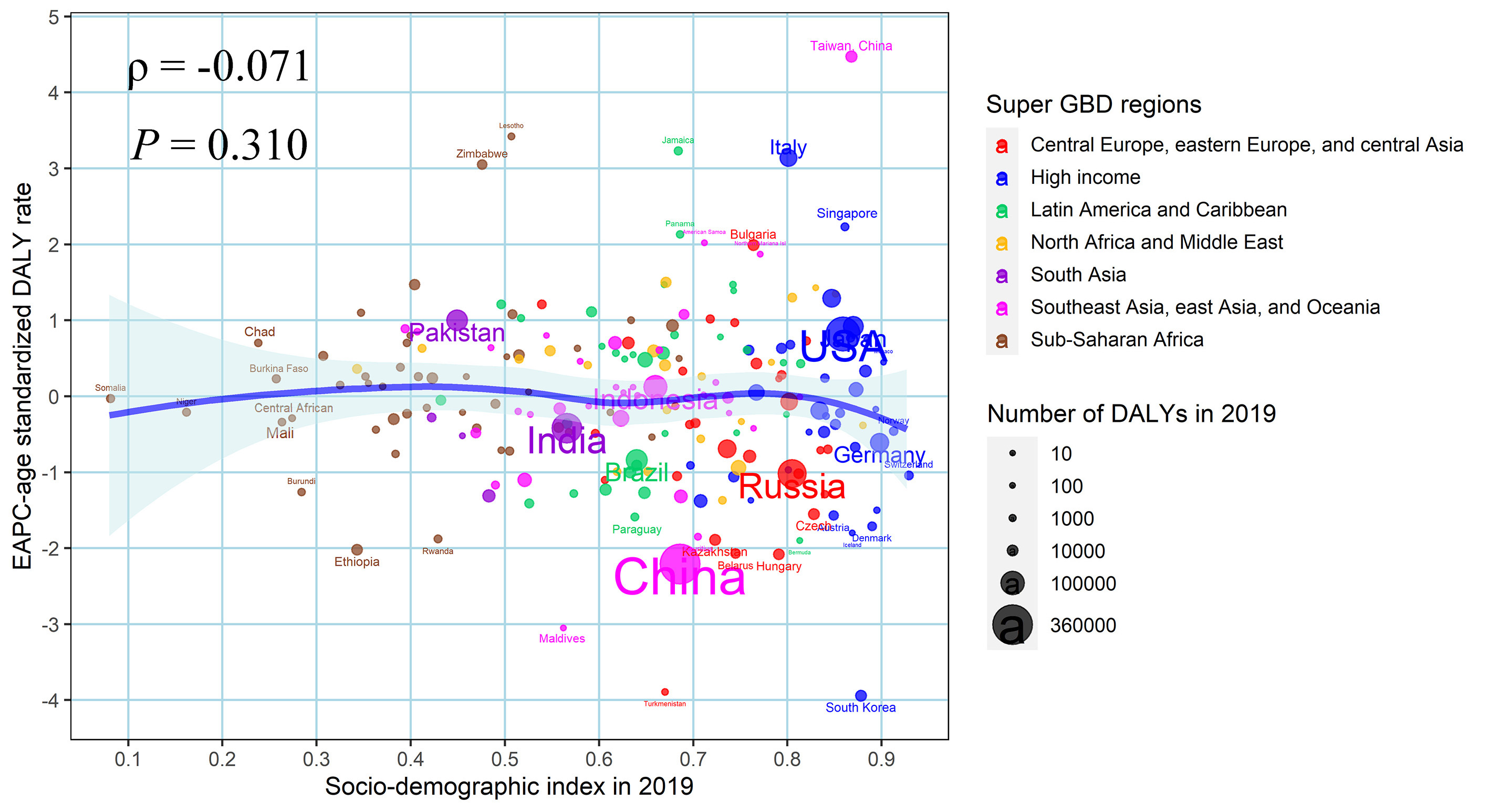

Supplement: Supplementary file 3 — Figure S3 [file CAM4-11-2467-s006.jpg]

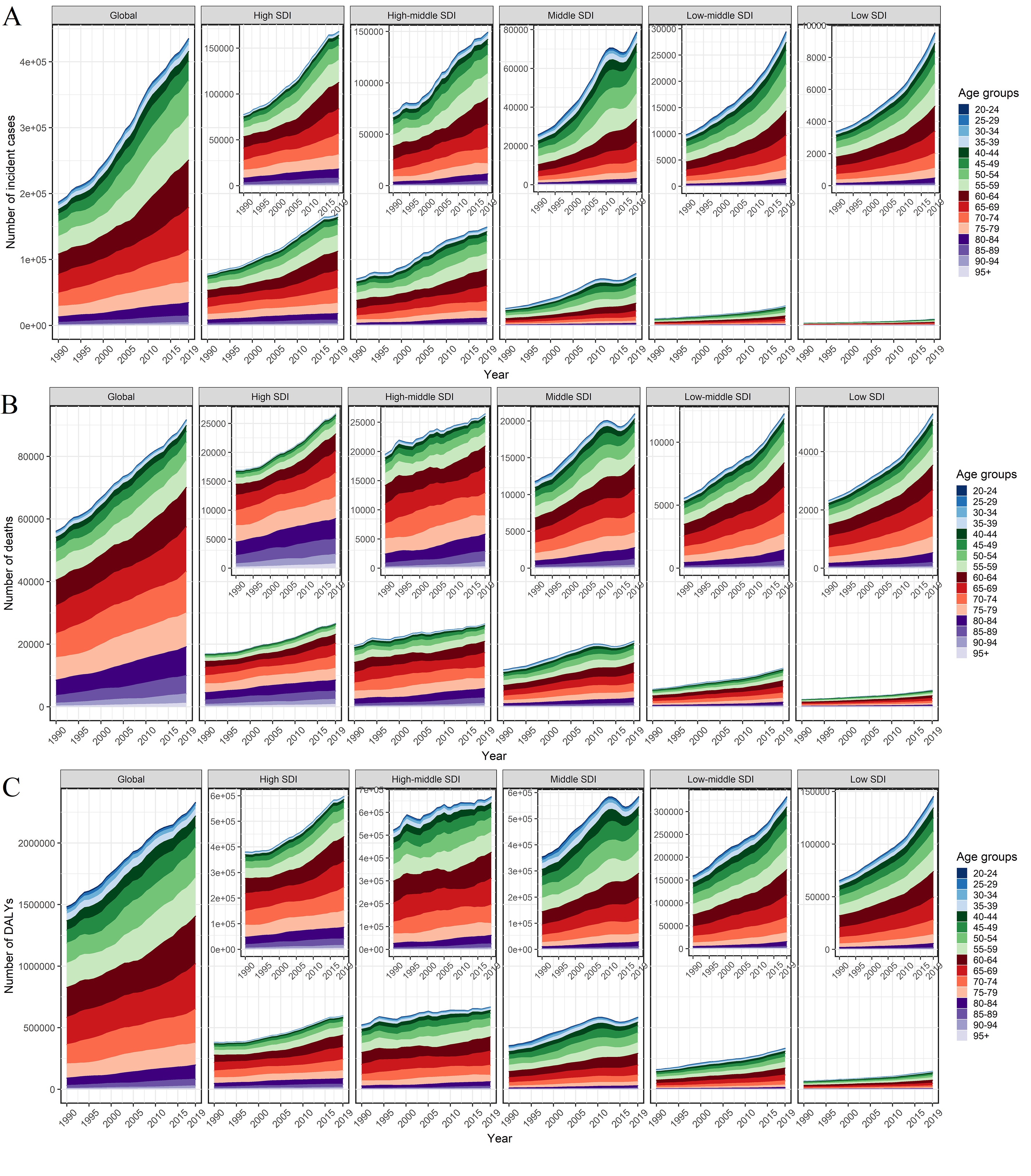

Supplement: Supplementary file 4 — Figure S4 [file CAM4-11-2467-s001.jpg]

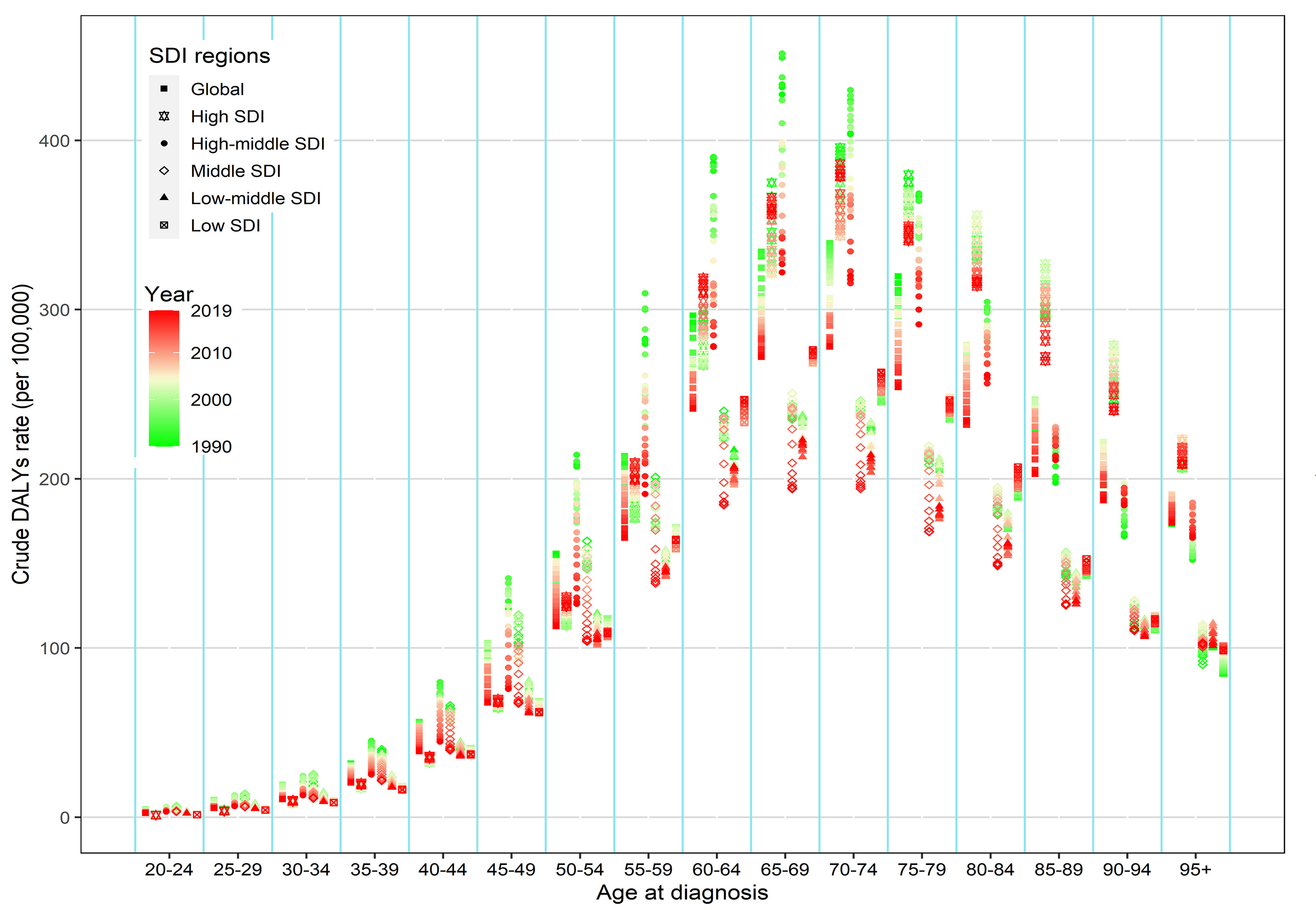

Supplement: Supplementary file 5 — Figure S5 [file CAM4-11-2467-s005.jpg]
