# Supplementary material for: Spatiotemporal trends in burden of uterine cancer and its attribution to body mass index in 204 countries and territories from 1990 to 2019
Source: Cancer Med. 2022 Feb 13;11(12):2467–81. doi: 10.1002/cam4.4608 (PMC9189473; doi:10.1002/cam4.4608)
Supplement: Supplementary file 6 — Table S1. Table S2. Table S3. Table S4. Table S5. Table S6. Table S7. Table S8. Table S9. [file CAM4-11-2467-s002.doc]

**Spatiotemporal trends in burden of uterine cancer and its attributable risk factors in 204 countries and territories from 1990 to 2019**

**9 supplementary tables and 5 supplementary figures.**

**Contents**

**Table S1.** Incident cases of uterine cancer and EAPC in ASIR by location, 1990–2019.

**Table S2.** Death cases of uterine cancer and EAPC in ASDR by location, 1990–2019.

**Table S3.** DALYs of uterine cancer and EAPC in age-standardized DALY rates by location, 1990–2019.

**Table S4.** Incident cases of uterine cancer and EAPC in ASIR by age, 1990–2019.

**Table S5.** Top 20 countries or territories with the highest ASIR in 2019.

**Table S6.** Top 20 countries or territories with the highest ASDR in 2019.

**Table S7.** Top 20 countries or territories with the most rapid increase in ASIR from 1990 to 2019.

**Table S8.** Top 20 countries or territories with the most rapid increase in ASDR from 1990 to 2019.

**Table S9.** The proportion of deaths from uterine cancer attributable to high BMI in 2019 and 1990.

**Figure S1.** The global disease burden of uterine cancer in 204 countries and territories. A: The death cases of uterine cancer in 2019, B: The age-standardized DALY rates of uterine cancer in 2019, and C: EAPC in the age-standardized DALY rates of uterine cancer in 2019. Note: DALY, disability-adjusted life year; EAPC, estimated annual percentage change.

**Figure S2.** The age-standardized DALY rate per 100000 person-years of uterine cancer for 21 GBD regions by SDI, 1990–2019. Expected values based on SDI and age-standardized rates in all locations are shown as the blue line. For each region, points from big to small depict estimates from each year from 1990 to 2019. Note: GBD: Global Burden of Diseases, Injuries, and Risk Factors Study. SDI: Socio-demographic Index. DALY: disability-adjusted life year.

**Figure S3.** The correlation between EAPC of the age-standardized DALY rate and SDI of 2019 in 204 countries or territories. The size of circle represents the quantity of uterine cancer patients in one country or territory. Note: DALY, disability-adjusted life year; EAPC, estimated annual percentage change; SDI, socio-demographic index.

**Figure S4.** The number of incident cases, deaths and DALYs of uterine cancer in different age groups in the globe and various SDI regions from 1990 to 2019. Note: SDI, socio-demographic index.

**Figure S5.** The crude DALY rates of uterine cancer in different SDI regions by age from 1990 to 2019. Note: DALY, disability-adjusted life year; EAPC, estimated annual percentage change; SDI, socio-demographic index.

**Table S1 Incident cases of uterine cancer and EAPC in ASIR by location, 1990–2019**

|  | **1990** | | **2019** | | **1990-2019** |
| --- | --- | --- | --- | --- | --- |
|  | **Incident cases**  **No.×10^3^ (95% UI)** | **ASIR per 100 000**  **No. (95% UI)** | **Incident cases**  **No.×10^3^ (95% UI)** | **ASIR per 100 000**  **No. (95% UI)** | **EAPC in ASIR**  **No. (95% CI)** |
| **Overall** | 187.19(174.63 ,196.03) | 8.67 (8.1 ,9.08) | 435.04(397.02 ,479.73) | 9.99 (9.12 ,11.02) | 0.69 (0.57 ,0.81) |
| **SDI region** |  |  |  |  |  |
| High SDI | 77.5 (74.62 ,79.41) | 13.82 (13.36 ,14.15) | 168.02(148.61 ,188.55) | 19.16 (16.94 ,21.48) | 1.37 (1.28 ,1.46) |
| High-middle SDI | 70.52 (67.44 ,73.69) | 11.66 (11.15 ,12.19) | 149.13(133.48 ,165.01) | 13.87 (12.4 ,15.37) | 0.8 (0.64 ,0.97) |
| Middle SDI | 25.82 (19.86 ,29.47) | 4.45 (3.48 ,5.05) | 78.64 (64.69 ,92.03) | 5.7 (4.72 ,6.67) | 1.1 (0.77 ,1.43) |
| Low-middle SDI | 9.85 (8.01 ,11.8) | 3.09 (2.53 ,3.71) | 29.42 (25.04 ,35.62) | 3.94 (3.36 ,4.8) | 0.73 (0.66 ,0.79) |
| Low SDI | 3.39 (2.69 ,4.23) | 2.78 (2.2 ,3.5) | 9.53 (7.77 ,11.74) | 3.43 (2.81 ,4.21) | 0.71 (0.65 ,0.77) |
| **GBD region** |  |  |  |  |  |
| High-income Asia Pacific | 7.42 (6.59 ,7.79) | 6.74 (5.95, 7.08) | 18 (14.66 ,21.64) | 11.32 (9.21, 13.68) | 2.46 (2.21,2.7) |
| High-income North America | 35.82 (34.36 ,36.83) | 19.15 (18.49, 19.68) | 86.65 (72.24 ,103.73) | 27.82 (23.11, 33.44) | 1.44 (1.34,1.53) |
| Western Europe | 40.04 (38.53 ,41.25) | 13.14 (12.68, 13.52) | 82.5 (70.94 ,94.18) | 19.62 (16.98, 22.47) | 1.71 (1.57,1.86) |
| Australasia | 1.16 (1.08 ,1.23) | 9.46 (8.88, 10.03) | 2.69 (2.18 ,3.32) | 11.26 (9.13, 13.94) | 0.58 (0.45,0.7) |
| Tropical Latin America | 2.97 (2.83 ,3.11) | 5.92 (5.62, 6.2) | 9.35 (8.72 ,10.02) | 6.97 (6.5, 7.48) | 0.45 (0.36,0.53) |
| Andean Latin America | 0.84 (0.67 ,0.97) | 7.57 (6.06, 8.74) | 2.9 (2.28 ,3.77) | 9.75 (7.65, 12.67) | 0.91 (0.76,1.05) |
| Central Latin America | 1.89 (1.82 ,1.96) | 4.1 (3.94, 4.26) | 8.37 (7.04 ,9.92) | 6.4 (5.39, 7.58) | 1.54 (1.36,1.71) |
| Southern Latin America | 1.77 (1.67 ,1.88) | 6.97 (6.59, 7.36) | 3.64 (2.86 ,4.59) | 8.23 (6.43, 10.38) | 0.32 (0.15,0.49) |
| Caribbean | 1.61 (1.5 ,1.72) | 11.61 (10.82, 12.43) | 4.84 (4.11 ,5.68) | 17.83 (15.11, 20.97) | 1.34 (1.1,1.57) |
| Eastern Europe | 34.57 (33.21 ,36.19) | 20.42 (19.56, 21.44) | 52.51 (44.7 ,61.83) | 27.5 (23.25, 32.58) | 0.91 (0.63,1.19) |
| Central Europe | 11.34 (10.91 ,11.94) | 13.83 (13.32, 14.55) | 21.86 (18.94 ,25.3) | 20.52 (17.68, 23.86) | 1.56 (1.47,1.65) |
| Central Asia | 3.05 (2.88 ,3.24) | 10.77 (10.18, 11.44) | 5.46 (4.86 ,6.11) | 11.72 (10.47, 13.11) | 0.36 (0.17,0.54) |
| North Africa and Middle East | 2.84 (2.15 ,3.4) | 3.1 (2.36, 3.73) | 12.51 (8.47 ,14.86) | 5.41 (3.71, 6.39) | 2.14 (1.84,2.43) |
| South Asia | 6.02 (4.71 ,7.59) | 2.17 (1.71, 2.74) | 21.83 (17.28 ,26.69) | 2.94 (2.34, 3.61) | 0.88 (0.71,1.06) |
| Southeast Asia | 7.14 (4.97 ,8.35) | 4.7 (3.36, 5.46) | 22.13 (14.28 ,26.65) | 6.23 (4.1, 7.47) | 0.9 (0.86,0.94) |
| East Asia | 25.44 (18.94 ,31.28) | 5.17 (3.92, 6.33) | 70.92 (55.04 ,96.59) | 6.55 (5.07, 8.8) | 1.35 (0.7,1.99) |
| Oceania | 0.11 (0.08 ,0.14) | 6.75 (4.79, 8.38) | 0.34 (0.19 ,0.46) | 8.58 (4.86, 11.31) | 0.86 (0.82,0.89) |
| Western Sub-Saharan Africa | 0.93 (0.75 ,1.36) | 2.13 (1.73, 3.16) | 2.68 (2.15 ,3.6) | 2.64 (2.13, 3.58) | 0.83 (0.77,0.89) |
| Eastern Sub-Saharan Africa | 1.3 (0.91 ,1.6) | 3.27 (2.32, 4.01) | 3.27 (2.36 ,4.08) | 3.7 (2.64, 4.57) | 0.38 (0.28,0.49) |
| Central Sub-Saharan Africa | 0.37 (0.27 ,0.52) | 2.82 (2.11, 3.96) | 0.92 (0.65 ,1.31) | 3.01 (2.14, 4.32) | 0.2 (0.1,0.3) |
| Southern Sub-Saharan Africa | 0.55 (0.45 ,0.65) | 3.48 (2.82, 4.05) | 1.68 (1.19 ,1.95) | 5.08 (3.57, 5.87) | 1.74 (1.55,1.94) |

Note: ASIR: age-standardized incidence rate; EAPC: the estimated annual percentage change; SDI: socio-demographic index

**Table S2 Death cases of uterine cancer and EAPC in ASDR by location, 1990–2019**

|  | **1990** | | **2019** | | **1990-2019** |
| --- | --- | --- | --- | --- | --- |
|  | **Deaths**  **No.×10^3^ (95% UI)** | **ASDR per 100 000**  **No. (95% UI)** | **Deaths**  **No.×10^3^ (95% UI)** | **ASDR per 100 000**  **No. (95% UI)** | **EAPC in ASDR**  **No. (95% CI)** |
| **Overall** | 56.13 (51.1 ,60.2) | 2.67 (2.44, 2.86) | 91.64 (82.39 ,101.5) | 2.09 (1.88, 2.32) | -0.85(-0.93,-0.76) |
| **SDI region** |  |  |  |  |  |
| High SDI | 16.88 (15.87 ,17.41) | 2.73 (2.57, 2.81) | 26.63 (24 ,28.14) | 2.52 (2.32, 2.64) | -0.12 (-0.2,-0.03) |
| High-middle SDI | 19.52 (18.33 ,20.67) | 3.23 (3.03, 3.42) | 26.43 (23.96 ,28.83) | 2.33 (2.12, 2.55) | -1.24(-1.36,-1.13) |
| Middle SDI | 11.81 (9.28 ,13.51) | 2.22 (1.77, 2.52) | 20.95 (17.53 ,24.33) | 1.61 (1.36, 1.87) | -1.01(-1.27,-0.75) |
| Low-middle SDI | 5.57 (4.59 ,6.75) | 1.93 (1.61, 2.36) | 12.25 (10.43 ,15.28) | 1.75 (1.49, 2.21) | -0.48(-0.57,-0.38) |
| Low SDI | 2.32 (1.84 ,2.93) | 2.08 (1.64, 2.65) | 5.3 (4.32 ,6.64) | 2.1 (1.72, 2.63) | -0.01 (-0.05,0.03) |
| **GBD region** |  |  |  |  |  |
| High-income Asia Pacific | 2.19 (1.94 ,2.3) | 1.94 (1.71, 2.04) | 3.43 (2.89 ,3.76) | 1.47 (1.3, 1.58) | -0.63(-0.84,-0.42) |
| High-income North America | 6.14 (5.75 ,6.35) | 2.92 (2.76, 3.01) | 11.26 (10.46 ,11.84) | 3.23 (3.03, 3.38) | 0.51 (0.38,0.63) |
| Western Europe | 9.11 (8.56 ,9.44) | 2.61 (2.46, 2.69) | 13.81 (12.22 ,14.71) | 2.59 (2.35, 2.74) | 0.2 (0.1,0.31) |
| Australasia | 0.35 (0.32 ,0.36) | 2.62 (2.46, 2.77) | 0.65 (0.57 ,0.72) | 2.4 (2.13, 2.65) | -0.34 (-0.4,-0.27) |
| Tropical Latin America | 1.42 (1.34 ,1.49) | 3.08 (2.89, 3.22) | 3.18 (2.93 ,3.42) | 2.4 (2.2, 2.57) | -0.96(-1.04,-0.87) |
| Andean Latin America | 0.47 (0.38 ,0.54) | 4.53 (3.67, 5.21) | 1.02 (0.81 ,1.34) | 3.53 (2.8, 4.63) | -0.85(-0.95,-0.74) |
| Central Latin America | 0.87 (0.83 ,0.91) | 2.08 (1.97, 2.17) | 2.34 (1.99 ,2.73) | 1.84 (1.57, 2.16) | -0.48 (-0.7,-0.27) |
| Southern Latin America | 0.77 (0.72 ,0.81) | 2.99 (2.82, 3.15) | 1.14 (1.04 ,1.25) | 2.4 (2.19, 2.62) | -1.01(-1.16,-0.86) |
| Caribbean | 0.61 (0.56 ,0.67) | 4.54 (4.18, 4.99) | 1.56 (1.34 ,1.82) | 5.68 (4.86, 6.62) | 0.8 (0.68,0.93) |
| Eastern Europe | 8.06 (7.72 ,8.39) | 4.45 (4.27, 4.64) | 8.44 (7.31 ,9.67) | 3.92 (3.39, 4.51) | -0.95(-1.22,-0.69) |
| Central Europe | 3.56 (3.42 ,3.77) | 4.23 (4.05, 4.47) | 4.72 (4.1 ,5.43) | 3.79 (3.28, 4.36) | -0.3 (-0.43,-0.18) |
| Central Asia | 1.11 (1.05 ,1.18) | 4.02 (3.8, 4.26) | 1.35 (1.21 ,1.51) | 3.2 (2.88, 3.57) | -0.76 (-0.9,-0.63) |
| North Africa and Middle East | 1.34 (1.02 ,1.62) | 1.62 (1.25, 1.99) | 3.23 (2.35 ,3.81) | 1.58 (1.17, 1.85) | 0.05 (-0.23,0.33) |
| South Asia | 3.88 (3.05 ,4.92) | 1.57 (1.23, 2.02) | 10.25 (8.13 ,13.08) | 1.47 (1.16, 1.87) | -0.44(-0.59,-0.29) |
| Southeast Asia | 3.2 (2.34 ,3.79) | 2.31 (1.73, 2.73) | 7.32 (5.13 ,8.65) | 2.21 (1.6, 2.6) | -0.22 (-0.3,-0.13) |
| East Asia | 10.89 (8.18 ,13.3) | 2.35 (1.8, 2.86) | 12.93 (10.12 ,17.99) | 1.19 (0.93, 1.65) | -2.16(-2.74,-1.57) |
| Oceania | 0.05 (0.04 ,0.06) | 3.52 (2.45, 4.39) | 0.15 (0.08 ,0.2) | 4.18 (2.44, 5.5) | 0.69 (0.63,0.74) |
| Western Sub-Saharan Africa | 0.64 (0.52 ,0.95) | 1.54 (1.24, 2.32) | 1.51 (1.22 ,2.05) | 1.66 (1.36, 2.27) | 0.38 (0.33,0.43) |
| Eastern Sub-Saharan Africa | 0.9 (0.65 ,1.12) | 2.48 (1.78, 3.09) | 1.92 (1.34 ,2.38) | 2.41 (1.7, 3.01) | -0.12 (-0.2,-0.04) |
| Central Sub-Saharan Africa | 0.25 (0.19 ,0.35) | 2.1 (1.59, 2.93) | 0.55 (0.39 ,0.78) | 1.97 (1.42, 2.8) | -0.23(-0.28,-0.18) |
| Southern Sub-Saharan Africa | 0.31 (0.25 ,0.37) | 2.09 (1.65, 2.48) | 0.87 (0.6 ,1) | 2.77 (1.92, 3.18) | 1.47 (1.17,1.77) |

Note: ASDR: age-standardized death rate; EAPC: the estimated annual percentage change; SDI: socio-demographic index

**Table S3 DALYs of uterine cancer and EAPC in age-standardized DALY rates by location, 1990–2019**

|  | **1990** |  | **2019** |  | **1990-2019** |
| --- | --- | --- | --- | --- | --- |
|  | **DALYs**  **No.×10^4^ (95% UI)** | **DALY per 100 000**  **No. (95% UI)** | **DALYs**  **No.×10^4^ (95% UI)** | **DALY per 100 000**  **No. (95% UI)** | **EAPC in DALY**  **No. (95% CI)** |
| **Overall** | 148.33(131.75 ,161.27) | 68.33 (60.86, 74.23) | 232.91(209.29 ,256.09) | 53.54 (48.13, 58.84) | -0.84(-0.93,-0.75) |
| **SDI region** |  |  |  |  |  |
| High SDI | 38.01 (36.17 ,39.56) | 66.52 (63.07, 69.26) | 59.68 (55.58 ,63.61) | 65.31 (60.99, 69.65) | 0.15 (0.04,0.26) |
| High-middle SDI | 52.32 (48.64 ,55.87) | 86.43 (80.17, 92.33) | 66.79 (60.62 ,73.52) | 61.5 (55.81, 67.85) | -1.3 (-1.43,-1.17) |
| Middle SDI | 35.42 (26.69 ,40.94) | 60.49 (46.35, 69.57) | 58.46 (47.58 ,67.48) | 42.88 (34.87, 49.47) | -1.1 (-1.37,-0.83) |
| Low-middle SDI | 15.94 (12.95 ,19.36) | 49.01 (39.98, 59.48) | 33.22 (28.09 ,40.49) | 44.53 (37.69, 54.57) | -0.48(-0.58,-0.37) |
| Low SDI | 6.54 (5.14 ,8.24) | 51.5 (40.84, 65.01) | 14.53 (11.79 ,18.15) | 51.24 (41.73, 64.02) | -0.07(-0.12,-0.03) |
| **GBD region** |  |  |  |  |  |
| High-income Asia Pacific | 5.58 (4.73 ,5.91) | 49.96 (42, 52.95) | 7.67 (6.89 ,8.35) | 42.38 (38.85, 45.96) | -0.11 (-0.35,0.13) |
| High-income North America | 13.98 (13.27 ,14.65) | 72.79 (69.27, 76.36) | 27.25 (25.41 ,29.1) | 85.62 (79.99, 91.48) | 0.75 (0.63,0.87) |
| Western Europe | 19.63 (18.65 ,20.48) | 62.16 (59.26, 64.85) | 28.12 (25.7 ,30.18) | 63.23 (58.28, 67.78) | 0.32 (0.22,0.42) |
| Australasia | 0.77 (0.73 ,0.81) | 61.71 (58.26, 65.17) | 1.38 (1.24 ,1.52) | 56.3 (50.79, 61.96) | -0.33 (-0.4,-0.26) |
| Tropical Latin America | 3.61 (3.45 ,3.77) | 71.87 (68.47, 75.08) | 7.66 (7.15 ,8.2) | 57.25 (53.41, 61.27) | -0.87(-0.96,-0.78) |
| Andean Latin America | 1.27 (1.02 ,1.46) | 113.36 (90.87, 130.54) | 2.53 (1.97 ,3.32) | 85.55 (66.72, 112.49) | -0.96(-1.07,-0.85) |
| Central Latin America | 2.32 (2.24 ,2.42) | 50.41 (48.5, 52.56) | 6.14 (5.21 ,7.21) | 47.36 (40.25, 55.58) | -0.24 (-0.49,0) |
| Southern Latin America | 1.86 (1.75 ,1.97) | 72.81 (68.69, 76.97) | 2.54 (2.31 ,2.79) | 56.68 (51.56, 62.19) | -1.07 (-1.25,-0.9) |
| Caribbean | 1.66 (1.53 ,1.83) | 119.52 (109.4, 131.55) | 3.97 (3.36 ,4.68) | 146.98(124.22, 173.54) | 0.7 (0.55,0.84) |
| Eastern Europe | 21.44 (20.53 ,22.54) | 124.15(118.61, 130.58) | 21.92 (19.02 ,25.25) | 110.79 (95.86, 128.31) | -0.93(-1.22,-0.64) |
| Central Europe | 8.96 (8.6 ,9.48) | 108.58(104.28, 114.78) | 10.75 (9.31 ,12.36) | 96.48 (83.29, 111.56) | -0.32(-0.45,-0.19) |
| Central Asia | 3.17 (3 ,3.36) | 112.56(106.46, 119.13) | 4.11 (3.66 ,4.63) | 89.73 (80.18, 100.92) | -0.82(-0.94,-0.71) |
| North Africa and Middle East | 3.77 (2.87 ,4.56) | 40.91 (31.33, 49.31) | 8.97 (6.27 ,10.75) | 39.33 (28.15, 46.59) | -0.05 (-0.33,0.23) |
| South Asia | 10.65 (8.35 ,13.42) | 36.89 (29.05, 46.71) | 27.05 (21.31 ,34.18) | 36.23 (28.51, 45.91) | -0.26 (-0.41,-0.11) |
| Southeast Asia | 9.61 (6.63 ,11.55) | 63.15 (45.15, 75) | 21.09 (13.75 ,25.14) | 59.84 (39.99, 71.06) | -0.26(-0.36,-0.15) |
| East Asia | 34.12 (24.48 ,42.17) | 68.79 (50.13, 84.6) | 38.6 (30.66 ,52.3) | 35.7 (28.39, 47.85) | -2.08 (-2.65,-1.5) |
| Oceania | 0.16 (0.11 ,0.2) | 94.6 (65.12, 118.8) | 0.46 (0.25 ,0.62) | 113.23 (63.01, 151.27) | 0.73 (0.66,0.8) |
| Western Sub-Saharan Africa | 1.67 (1.35 ,2.4) | 37.38 (30.15, 54.05) | 3.96 (3.17 ,5.32) | 38.46 (31.19, 51.7) | 0.16 (0.12,0.21) |
| Eastern Sub-Saharan Africa | 2.55 (1.8 ,3.19) | 61.55 (43.74, 76.34) | 5.13 (3.65 ,6.42) | 56.64 (40.16, 70.28) | -0.35(-0.43,-0.26) |
| Central Sub-Saharan Africa | 0.73 (0.55 ,1.01) | 53.4 (40.06, 73.87) | 1.53 (1.09 ,2.19) | 48.58 (34.5, 69.88) | -0.34 (-0.39,-0.3) |
| Southern Sub-Saharan Africa | 0.79 (0.64 ,0.94) | 49.39 (39.85, 58.05) | 2.08 (1.48 ,2.43) | 62.63 (44.04, 73.04) | 1.4 (1.11,1.69) |

Note: DALY: disability-adjusted life year; EAPC: the estimated annual percentage change; SDI: socio-demographic index

**Table S4 Incident cases of uterine cancer and EAPC in ASIR by ages, 1990–2019**

|  | **1990** |  | **2019** |  | **1990-2019** |
| --- | --- | --- | --- | --- | --- |
|  | **Incident cases**  **No.×10^3^ (95% UI)** | **ASIR per 100 000**  **No. (95% UI)** | **Incident cases**  **No.×10^3^ (95% UI)** | **ASIR per 100 000**  **No. (95% UI)** | **EAPC in ASIR**  **No. (95% CI)** |
| **Age groups** |  |  |  |  |  |
| 0-14 | 0 | 0 | 0 | 0 | 0 (0 ,0) |
| 15 to 19 | 0 (0 ,0) | 0 (0 ,0) | 0 (0 ,0) | 0 (0 ,0) | 0 (0 ,0) |
| 20 to 24 | 0.92 (0.6 ,1.09) | 0.38 (0.25 ,0.45) | 1.12 (0.77 ,1.32) | 0.38 (0.26 ,0.45) | -0.01 (-0.31 ,0.29) |
| 25 to 29 | 1.77 (1.29 ,2.04) | 0.81 (0.59 ,0.93) | 2.7 (2.06 ,3.12) | 0.9 (0.68 ,1.04) | 0.19 (-0.15 ,0.53) |
| 30 to 34 | 3.09 (2.44 ,3.47) | 1.62 (1.28 ,1.83) | 5.79 (4.56 ,6.67) | 1.94 (1.53 ,2.23) | 0.37 (0.11 ,0.63) |
| 35 to 39 | 5.55 (4.56 ,6.18) | 3.2 (2.62 ,3.56) | 9.81 (8.13 ,11.11) | 3.65 (3.03 ,4.14) | 0.55 (0.34 ,0.77) |
| 40 to 44 | 7.28 (6.27 ,7.98) | 5.2 (4.47 ,5.69) | 15.12 (12.95 ,16.98) | 6.18 (5.29 ,6.94) | 0.78 (0.49 ,1.07) |
| 45 to 49 | 11.61 (10.14 ,12.5) | 10.2 (8.9 ,10.97) | 29.05 (25.78 ,32.51) | 12.34 (10.95 ,13.81) | 0.7 (0.36 ,1.04) |
| 50 to 54 | 21.89 (19.88 ,23.43) | 20.86 (18.94 ,22.32) | 53.3 (47.55 ,60.01) | 24.31 (21.69 ,27.38) | 0.91 (0.63 ,1.18) |
| 55 to 59 | 26.71 (24.68 ,28.43) | 28.91 (26.72 ,30.77) | 67.22 (60.75 ,74.99) | 35.71 (32.27 ,39.84) | 1.11 (0.89 ,1.32) |
| 60 to 64 | 31 (29.55 ,32.51) | 37.78 (36.01 ,39.61) | 72.32 (65.59 ,79.6) | 45.09 (40.9 ,49.64) | 0.98 (0.8 ,1.16) |
| 65 to 69 | 28.3 (27.07 ,29.73) | 42.73 (40.87 ,44.9) | 64.19 (58.68 ,70.72) | 47.56 (43.48 ,52.4) | 0.6 (0.43 ,0.77) |
| 70 to 74 | 20.06 (19.09 ,21.05) | 42.7 (40.63 ,44.8) | 48.2 (43.86 ,53.36) | 48.69 (44.31 ,53.91) | 0.32 (0.22 ,0.43) |
| 75 to 79 | 15.97 (14.99 ,16.69) | 44.07 (41.36 ,46.04) | 31.55 (28.18 ,35.15) | 45.17 (40.34 ,50.33) | 0.19 (0.09 ,0.29) |
| 80 to 84 | 8.46 (7.57 ,8.98) | 38.38 (34.36 ,40.76) | 20.16 (16.92 ,22.8) | 41 (34.41 ,46.36) | 0.2 (0.01 ,0.39) |
| 85 to 89 | 3.47 (2.96 ,3.75) | 34.36 (29.3 ,37.1) | 9.94 (7.86 ,11.32) | 36.55 (28.89 ,41.63) | 0.32 (0.06 ,0.58) |
| 90 to 94 | 0.88 (0.71 ,0.97) | 27.92 (22.43 ,30.77) | 3.51 (2.68 ,4.03) | 30.4 (23.17 ,34.92) | 0.43 (0.18 ,0.68) |
| 95+ | 0.2 (0.16 ,0.23) | 26.4 (20.23 ,29.64) | 1.07 (0.79 ,1.24) | 30.63 (22.54 ,35.41) | 0.72 (0.55 ,0.89) |

Note: ASIR: age-standardized incidence rate; EAPC: the estimated annual percentage change

**Table S5 Top 20 countries or territories with the highest ASIR in 2019.**

| **Number** | **Location** | **ASIR per 100 000**  **No. (95% UI) in 2019** |
| --- | --- | --- |
| **1** | Northern Mariana Islands | 32.77(21.30,42.36) |
| **2** | Russia | 32.55(26.58,39.65) |
| **3** | Bulgaria | 30.66(23.26,39.76) |
| **4** | American Samoa | 29.82(19.30,39.04) |
| **5** | Grenada | 29.41(25.01,33.96) |
| **6** | Latvia | 29.10(21.51,39.10) |
| **7** | USA | 28.80(23.50,34.93) |
| **8** | Croatia | 27.85(20.93,36.12) |
| **9** | Italy | 26.93(20.70,34.54) |
| **10** | Cuba | 26.49(21.22,33.22) |
| **11** | North Macedonia | 25.12(17.39,32.84) |
| **12** | Estonia | 25.08(18.71,32.97) |
| **13** | Barbados | 24.28(19.58,29.57) |
| **14** | Georgia | 23.68(18.78,29.09) |
| **15** | Serbia | 23.04(15.40,30.59) |
| **16** | Netherlands | 23.02(17.48,29.53) |
| **17** | Andorra | 22.39(15.29,31.94) |
| **18** | Slovakia | 22.01(15.39,29.56) |
| **19** | Spain | 22.01(16.66,27.99) |
| **20** | Luxembourg | 21.75(17.04,26.98) |

Note: ASIR: age-standardized incidence rate

**Table S6 Top 20 countries or territories with the highest ASDR in 2019.**

| **Number** | **Location** | **ASDR per 100 000**  **No. (95% UI) in 2019** |
| --- | --- | --- |
| **1** | Grenada | 11.30(9.79,12.99) |
| **2** | American Samoa | 10.74(7.18,13.86) |
| **3** | Saint Vincent and the Grenadines | 8.08(7.02,9.29) |
| **4** | Northern Mariana Islands | 7.66(5.10,9.70) |
| **5** | Barbados | 7.55(6.22,9.01) |
| **6** | Solomon Islands | 7.53(4.16,10.46) |
| **7** | Jamaica | 7.04(5.58,8.76) |
| **8** | Micronesia | 7.02(3.61,10.38) |
| **9** | Guyana | 6.90(5.36,8.74) |
| **10** | Nauru | 6.88(3.26,9.60) |
| **11** | Marshall Islands | 6.66(3.55,9.65) |
| **12** | Saint Kitts and Nevis | 6.63(5.44,8.03) |
| **13** | Cuba | 6.48(5.30,8.06) |
| **14** | Trinidad and Tobago | 6.38(4.84,8.27) |
| **15** | Haiti | 5.80(3.82,8.36) |
| **16** | Georgia | 5.80(4.65,7.03) |
| **17** | Tokelau | 5.55(2.73,7.83) |
| **18** | Dominican Republic | 5.51(3.67,7.51) |
| **19** | Zimbabwe | 5.49(3.33,7.51) |
| **20** | Tuvalu | 5.47(2.82,7.96) |

Note: ASDR: age-standardized death rate.

**Table S7. Top 20 countries or territories with the most rapid increase in ASIR from 1990 to 2019.**

| **Number** | **Location** | **ASIR per 100 000 in 1990** | **ASIR per 100 000 in 2019** | **EAPC in ASIR**  **No. (95% CI)** |
| --- | --- | --- | --- | --- |
| **1** | Taiwan (Province of China) | 3.73 | 14.49 | 6.57 (6.02,7.13) |
| **2** | Italy | 8.92 | 26.93 | 4.81 (4.1,5.53) |
| **3** | Saudi Arabia | 2.07 | 6.73 | 4.76 (4.47,5.05) |
| **4** | Singapore | 3.79 | 9.93 | 4.46 (4.07,4.85) |
| **5** | Qatar | 4.03 | 9.49 | 4.17 (3.49,4.85) |
| **6** | Jamaica | 6.93 | 18.50 | 3.57 (3.07,4.07) |
| **7** | Lesotho | 2.83 | 5.76 | 3.53 (3.08,3.98) |
| **8** | Panama | 3.99 | 9.74 | 3.49 (3.32,3.65) |
| **9** | Bosnia and Herzegovina | 6.82 | 15.71 | 3.48 (2.96,4) |
| **10** | Iraq | 3.02 | 6.74 | 3.44 (2.9,3.98) |
| **11** | Japan | 6.70 | 14.51 | 3.38 (3.13,3.64) |
| **12** | Nicaragua | 2.20 | 5.12 | 3.16 (2.81,3.51) |
| **13** | North Macedonia | 11.37 | 25.12 | 3.05 (2.68,3.41) |
| **14** | Kuwait | 6.28 | 10.03 | 2.95 (2.27,3.64) |
| **15** | Bulgaria | 21.16 | 30.66 | 2.88 (2.09,3.68) |
| **16** | Sri Lanka | 3.47 | 6.53 | 2.81 (2.61,3.02) |
| **17** | Ireland | 12.81 | 21.12 | 2.74 (2.38,3.11) |
| **18** | Chile | 4.04 | 7.96 | 2.69 (2.49,2.89) |
| **19** | Honduras | 5.02 | 10.23 | 2.61 (2.37,2.84) |
| **20** | Serbia | 11.55 | 23.04 | 2.6 (2.37,2.83) |

Note: ASIR: age-standardized incidence rate; EAPC: the estimated annual percentage change.

**Table S8. Top 20 countries or territories with the most rapid increase in ASDR from 1990 to 2019.**

| **Number** | **Location** | **ASDR per 100 000 in 1990** | **ASDR per 100 000 in 2019** | **EAPC in ASDR**  **No. (95% CI)** |
| --- | --- | --- | --- | --- |
| **1** | Taiwan (Province of China) | 0.96 | 1.64 | 3.38 (2.92,3.85) |
| **2** | Lesotho | 2.07 | 3.91 | 3.27 (2.81,3.74) |
| **3** | Jamaica | 3.00 | 7.04 | 3.21 (2.83,3.6) |
| **4** | Italy | 1.29 | 2.24 | 2.72 (2.24,3.2) |
| **5** | Zimbabwe | 3.69 | 5.49 | 2.69 (2.06,3.33) |
| **6** | Qatar | 1.84 | 2.51 | 2.07 (1.52,2.62) |
| **7** | Panama | 1.77 | 2.68 | 2 (1.78,2.21) |
| **8** | Bulgaria | 4.51 | 5.10 | 1.98 (1.28,2.69) |
| **9** | Singapore | 1.41 | 1.92 | 1.96 (1.66,2.26) |
| **10** | American Samoa | 7.15 | 10.74 | 1.93 (1.68,2.17) |
| **11** | Northern Mariana Islands | 5.58 | 7.66 | 1.91 (1.49,2.32) |
| **12** | Kuwait | 2.01 | 2.21 | 1.7 (1,2.4) |
| **13** | Grenada | 7.46 | 11.30 | 1.64 (1.42,1.86) |
| **14** | Antigua and Barbuda | 3.10 | 5.43 | 1.62 (1.36,1.87) |
| **15** | Honduras | 2.86 | 4.33 | 1.6 (1.33,1.87) |
| **16** | Barbados | 5.26 | 7.55 | 1.6 (1.23,1.97) |
| **17** | Uganda | 2.64 | 4.08 | 1.59 (1.36,1.81) |
| **18** | Tajikistan | 2.68 | 3.91 | 1.5 (1.32,1.68) |
| **19** | UK | 2.58 | 3.23 | 1.37 (1.14,1.59) |
| **20** | Iraq | 1.45 | 1.92 | 1.33 (0.98,1.69) |

Note: ASDR: age-standardized death rate; EAPC: the estimated annual percentage change.

**Table S9. the proportion of deaths from uterine cancer attributable to high BMI in 2019 and 1990**

| **Location** | **Proportion of deaths from uterine cancer attributable to high BMI** | | | |
| --- | --- | --- | --- | --- |
| **Qatar** | 2019 | 66.07% | 1990 | 57.42% |
| **United Arab Emirates** | 2019 | 65.55% | 1990 | 54.38% |
| **Saudi Arabia** | 2019 | 63.64% | 1990 | 44.47% |
| **Kuwait** | 2019 | 63.59% | 1990 | 55.87% |
| **Jordan** | 2019 | 60.46% | 1990 | 51.11% |
| **Egypt** | 2019 | 59.46% | 1990 | 47.50% |
| **Bahrain** | 2019 | 59.43% | 1990 | 53.16% |
| **South Africa** | 2019 | 58.87% | 1990 | 52.37% |
| **Eswatini** | 2019 | 58.39% | 1990 | 52.12% |
| **American Samoa** | 2019 | 57.97% | 1990 | 57.54% |
| **Oman** | 2019 | 57.64% | 1990 | 32.56% |
| **Libya** | 2019 | 57.38% | 1990 | 49.19% |
| **Botswana** | 2019 | 57.20% | 1990 | 33.73% |
| **United States Virgin Islands** | 2019 | 56.73% | 1990 | 49.62% |
| **Turkey** | 2019 | 56.25% | 1990 | 48.59% |
| **Kazakhstan** | 2019 | 56.00% | 1990 | 49.34% |
| **Puerto Rico** | 2019 | 55.07% | 1990 | 47.93% |
| **Russian Federation** | 2019 | 54.87% | 1990 | 46.61% |
| **Ukraine** | 2019 | 54.87% | 1990 | 50.71% |
| **Belize** | 2019 | 54.62% | 1990 | 37.50% |
| **Northern Mariana Islands** | 2019 | 54.25% | 1990 | 55.76% |
| **United States of America** | 2019 | 54.20% | 1990 | 45.24% |
| **Estonia** | 2019 | 54.12% | 1990 | 44.20% |
| **Republic of Moldova** | 2019 | 54.12% | 1990 | 44.99% |
| **Latvia** | 2019 | 53.59% | 1990 | 47.59% |
| **Slovakia** | 2019 | 53.45% | 1990 | 47.76% |
| **Ecuador** | 2019 | 53.41% | 1990 | 43.84% |
| **Algeria** | 2019 | 53.37% | 1990 | 41.98% |
| **Trinidad and Tobago** | 2019 | 53.35% | 1990 | 43.92% |
| **Montenegro** | 2019 | 53.24% | 1990 | 50.03% |
| **Fiji** | 2019 | 53.20% | 1990 | 47.62% |
| **Romania** | 2019 | 53.17% | 1990 | 46.68% |
| **Lebanon** | 2019 | 53.03% | 1990 | 44.30% |
| **Czechia** | 2019 | 52.95% | 1990 | 44.12% |
| **Azerbaijan** | 2019 | 52.82% | 1990 | 41.29% |
| **Poland** | 2019 | 52.64% | 1990 | 46.10% |
| **Cook Islands** | 2019 | 52.48% | 1990 | 48.05% |
| **Bermuda** | 2019 | 52.40% | 1990 | 47.90% |
| **Lithuania** | 2019 | 52.39% | 1990 | 45.92% |
| **Serbia** | 2019 | 52.38% | 1990 | 47.11% |
| **Hungary** | 2019 | 52.36% | 1990 | 48.63% |
| **Syrian Arab Republic** | 2019 | 52.29% | 1990 | 43.53% |
| **Australia** | 2019 | 52.20% | 1990 | 41.53% |
| **Turkmenistan** | 2019 | 52.17% | 1990 | 41.33% |
| **Armenia** | 2019 | 51.99% | 1990 | 40.21% |
| **Belarus** | 2019 | 51.80% | 1990 | 44.19% |
| **Palau** | 2019 | 51.51% | 1990 | 46.76% |
| **Lesotho** | 2019 | 51.33% | 1990 | 32.52% |
| **Guam** | 2019 | 51.30% | 1990 | 44.82% |
| **Iraq** | 2019 | 51.18% | 1990 | 49.23% |
| **Bahamas** | 2019 | 50.85% | 1990 | 45.02% |
| **Tunisia** | 2019 | 50.82% | 1990 | 41.49% |
| **Jamaica** | 2019 | 50.81% | 1990 | 38.59% |
| **North Macedonia** | 2019 | 50.80% | 1990 | 45.48% |
| **Mexico** | 2019 | 50.72% | 1990 | 42.61% |
| **Croatia** | 2019 | 50.49% | 1990 | 43.65% |
| **Canada** | 2019 | 50.30% | 1990 | 40.65% |
| **Slovenia** | 2019 | 50.26% | 1990 | 46.07% |
| **Dominica** | 2019 | 50.05% | 1990 | 39.01% |
| **Morocco** | 2019 | 49.78% | 1990 | 35.93% |
| **Ghana** | 2019 | 49.66% | 1990 | 19.93% |
| **Barbados** | 2019 | 49.61% | 1990 | 41.60% |
| **Saint Kitts and Nevis** | 2019 | 49.47% | 1990 | 38.80% |
| **Bulgaria** | 2019 | 49.22% | 1990 | 48.88% |
| **Micronesia (Federated States of)** | 2019 | 49.04% | 1990 | 42.55% |
| **Tonga** | 2019 | 48.96% | 1990 | 46.73% |
| **Bosnia and Herzegovina** | 2019 | 48.82% | 1990 | 39.51% |
| **Greenland** | 2019 | 48.80% | 1990 | 42.88% |
| **Chile** | 2019 | 48.75% | 1990 | 39.27% |
| **Gabon** | 2019 | 48.69% | 1990 | 29.51% |
| **Niue** | 2019 | 48.62% | 1990 | 40.26% |
| **Iran (Islamic Republic of)** | 2019 | 48.61% | 1990 | 37.63% |
| **Nauru** | 2019 | 48.56% | 1990 | 46.36% |
| **Uzbekistan** | 2019 | 48.52% | 1990 | 37.87% |
| **Cameroon** | 2019 | 48.51% | 1990 | 42.26% |
| **Bolivia (Plurinational State of)** | 2019 | 48.31% | 1990 | 33.79% |
| **Brazil** | 2019 | 47.96% | 1990 | 34.30% |
| **Saint Lucia** | 2019 | 47.81% | 1990 | 34.86% |
| **Palestine** | 2019 | 47.53% | 1990 | 39.58% |
| **Georgia** | 2019 | 47.41% | 1990 | 45.44% |
| **Mauritania** | 2019 | 47.25% | 1990 | 35.09% |
| **Namibia** | 2019 | 47.00% | 1990 | 34.43% |
| **Monaco** | 2019 | 46.88% | 1990 | 43.40% |
| **Albania** | 2019 | 46.87% | 1990 | 35.92% |
| **Equatorial Guinea** | 2019 | 46.56% | 1990 | 11.74% |
| **Samoa** | 2019 | 46.54% | 1990 | 46.21% |
| **Suriname** | 2019 | 46.51% | 1990 | 35.63% |
| **Cuba** | 2019 | 46.32% | 1990 | 38.31% |
| **Sudan** | 2019 | 46.26% | 1990 | 28.43% |
| **Grenada** | 2019 | 46.15% | 1990 | 27.60% |
| **Saint Vincent and the Grenadines** | 2019 | 46.00% | 1990 | 28.94% |
| **El Salvador** | 2019 | 45.91% | 1990 | 34.39% |
| **Spain** | 2019 | 45.71% | 1990 | 40.89% |
| **Guyana** | 2019 | 45.61% | 1990 | 32.39% |
| **Kyrgyzstan** | 2019 | 44.96% | 1990 | 38.77% |
| **New Zealand** | 2019 | 44.84% | 1990 | 37.46% |
| **Tokelau** | 2019 | 44.82% | 1990 | 33.69% |
| **Peru** | 2019 | 44.81% | 1990 | 32.16% |
| **United Kingdom** | 2019 | 44.73% | 1990 | 38.73% |
| **Antigua and Barbuda** | 2019 | 44.56% | 1990 | 29.30% |
| **Iceland** | 2019 | 44.52% | 1990 | 40.57% |
| **Nicaragua** | 2019 | 44.45% | 1990 | 32.15% |
| **San Marino** | 2019 | 44.26% | 1990 | 41.34% |
| **Israel** | 2019 | 44.25% | 1990 | 39.11% |
| **Germany** | 2019 | 44.18% | 1990 | 39.22% |
| **Congo** | 2019 | 44.11% | 1990 | 26.84% |
| **Panama** | 2019 | 44.08% | 1990 | 24.03% |
| **Colombia** | 2019 | 44.03% | 1990 | 32.56% |
| **Costa Rica** | 2019 | 43.96% | 1990 | 36.03% |
| **Venezuela (Bolivarian Republic of)** | 2019 | 43.90% | 1990 | 37.33% |
| **Argentina** | 2019 | 43.65% | 1990 | 31.55% |
| **Andorra** | 2019 | 43.62% | 1990 | 41.06% |
| **Uruguay** | 2019 | 43.32% | 1990 | 32.72% |
| **Ireland** | 2019 | 43.17% | 1990 | 36.43% |
| **Liberia** | 2019 | 42.84% | 1990 | 32.78% |
| **Paraguay** | 2019 | 42.74% | 1990 | 34.33% |
| **Sao Tome and Principe** | 2019 | 42.74% | 1990 | 28.14% |
| **Zimbabwe** | 2019 | 42.65% | 1990 | 34.92% |
| **Greece** | 2019 | 42.58% | 1990 | 37.67% |
| **Austria** | 2019 | 41.84% | 1990 | 35.19% |
| **Luxembourg** | 2019 | 41.64% | 1990 | 37.45% |
| **Sweden** | 2019 | 41.50% | 1990 | 34.14% |
| **France** | 2019 | 41.38% | 1990 | 32.87% |
| **Finland** | 2019 | 41.23% | 1990 | 35.58% |
| **Kiribati** | 2019 | 41.15% | 1990 | 39.14% |
| **Belgium** | 2019 | 40.76% | 1990 | 34.60% |
| **Dominican Republic** | 2019 | 40.67% | 1990 | 26.29% |
| **Honduras** | 2019 | 40.46% | 1990 | 26.49% |
| **Mongolia** | 2019 | 40.28% | 1990 | 31.81% |
| **Mauritius** | 2019 | 39.95% | 1990 | 29.13% |
| **Benin** | 2019 | 39.86% | 1990 | 23.51% |
| **Malta** | 2019 | 39.81% | 1990 | 32.11% |
| **Italy** | 2019 | 39.66% | 1990 | 34.87% |
| **Cabo Verde** | 2019 | 39.59% | 1990 | 22.58% |
| **Netherlands** | 2019 | 39.50% | 1990 | 35.76% |
| **Afghanistan** | 2019 | 39.46% | 1990 | 28.02% |
| **Portugal** | 2019 | 39.46% | 1990 | 32.31% |
| **Denmark** | 2019 | 39.45% | 1990 | 31.60% |
| **Tuvalu** | 2019 | 39.44% | 1990 | 29.54% |
| **Vanuatu** | 2019 | 39.31% | 1990 | 33.94% |
| **Switzerland** | 2019 | 39.12% | 1990 | 34.59% |
| **Marshall Islands** | 2019 | 38.82% | 1990 | 27.04% |
| **Malaysia** | 2019 | 38.77% | 1990 | 23.83% |
| **Seychelles** | 2019 | 38.73% | 1990 | 32.57% |
| **Solomon Islands** | 2019 | 37.50% | 1990 | 29.62% |
| **United Republic of Tanzania** | 2019 | 36.70% | 1990 | 22.67% |
| **Gambia** | 2019 | 36.54% | 1990 | 22.75% |
| **Senegal** | 2019 | 36.42% | 1990 | 25.63% |
| **Guatemala** | 2019 | 36.21% | 1990 | 21.53% |
| **Norway** | 2019 | 36.17% | 1990 | 30.17% |
| **Togo** | 2019 | 36.06% | 1990 | 21.00% |
| **Cyprus** | 2019 | 35.79% | 1990 | 27.62% |
| **Côte d'Ivoire** | 2019 | 35.38% | 1990 | 26.74% |
| **South Sudan** | 2019 | 35.24% | 1990 | 22.79% |
| **Zambia** | 2019 | 34.93% | 1990 | 19.18% |
| **Thailand** | 2019 | 34.85% | 1990 | 16.47% |
| **Burkina Faso** | 2019 | 34.49% | 1990 | 14.20% |
| **Kenya** | 2019 | 34.24% | 1990 | 20.83% |
| **Sri Lanka** | 2019 | 33.47% | 1990 | 19.66% |
| **Nigeria** | 2019 | 32.77% | 1990 | 18.55% |
| **Guinea** | 2019 | 32.47% | 1990 | 22.06% |
| **Bhutan** | 2019 | 32.36% | 1990 | 15.95% |
| **Tajikistan** | 2019 | 31.60% | 1990 | 23.70% |
| **Mali** | 2019 | 31.44% | 1990 | 15.88% |
| **Yemen** | 2019 | 31.35% | 1990 | 19.01% |
| **Indonesia** | 2019 | 31.26% | 1990 | 11.01% |
| **Singapore** | 2019 | 30.85% | 1990 | 17.00% |
| **Taiwan (Province of China)** | 2019 | 30.19% | 1990 | 23.50% |
| **Pakistan** | 2019 | 29.93% | 1990 | 14.08% |
| **Uganda** | 2019 | 29.42% | 1990 | 11.66% |
| **Philippines** | 2019 | 29.33% | 1990 | 16.88% |
| **Guinea-Bissau** | 2019 | 29.22% | 1990 | 18.23% |
| **Sierra Leone** | 2019 | 29.20% | 1990 | 16.76% |
| **Rwanda** | 2019 | 28.78% | 1990 | 14.93% |
| **Comoros** | 2019 | 28.72% | 1990 | 20.00% |
| **Malawi** | 2019 | 28.31% | 1990 | 12.41% |
| **Djibouti** | 2019 | 28.18% | 1990 | 10.09% |
| **Maldives** | 2019 | 27.56% | 1990 | 11.77% |
| **Brunei Darussalam** | 2019 | 27.55% | 1990 | 15.46% |
| **Angola** | 2019 | 27.02% | 1990 | 9.08% |
| **Papua New Guinea** | 2019 | 26.81% | 1990 | 22.77% |
| **Lao People's Democratic Republic** | 2019 | 26.64% | 1990 | 10.42% |
| **Madagascar** | 2019 | 26.59% | 1990 | 15.06% |
| **India** | 2019 | 26.58% | 1990 | 10.85% |
| **Myanmar** | 2019 | 26.44% | 1990 | 8.64% |
| **Niger** | 2019 | 25.75% | 1990 | 17.77% |
| **Eritrea** | 2019 | 25.56% | 1990 | 10.50% |
| **Mozambique** | 2019 | 25.24% | 1990 | 9.67% |
| **Republic of Korea** | 2019 | 24.72% | 1990 | 19.29% |
| **China** | 2019 | 24.19% | 1990 | 11.57% |
| **Chad** | 2019 | 23.83% | 1990 | 11.46% |
| **Nepal** | 2019 | 22.26% | 1990 | 7.12% |
| **Haiti** | 2019 | 21.99% | 1990 | 15.85% |
| **Democratic Republic of the Congo** | 2019 | 20.72% | 1990 | 21.73% |
| **Ethiopia** | 2019 | 19.47% | 1990 | 8.51% |
| **Cambodia** | 2019 | 18.91% | 1990 | 8.81% |
| **Japan** | 2019 | 18.70% | 1990 | 17.91% |
| **Central African Republic** | 2019 | 18.16% | 1990 | 12.74% |
| **Viet Nam** | 2019 | 18.11% | 1990 | 6.39% |
| **Burundi** | 2019 | 18.07% | 1990 | 12.32% |
| **Bangladesh** | 2019 | 16.08% | 1990 | 4.87% |
| **Timor-Leste** | 2019 | 11.51% | 1990 | 8.42% |
| **Somalia** | 2019 | 10.77% | 1990 | 9.61% |
| **Democratic People's Republic of Korea** | 2019 | 10.35% | 1990 | 9.86% |
